# A Novel Pathogenicity Gene Is Required in the Rice Blast Fungus to Suppress the Basal Defenses of the Host

**DOI:** 10.1371/journal.ppat.1000401

**Published:** 2009-04-24

**Authors:** Myoung-Hwan Chi, Sook-Young Park, Soonok Kim, Yong-Hwan Lee

**Affiliations:** Department of Agricultural Biotechnology, Center for Fungal Genetic Resources, and Center for Fungal Pathogenesis, Seoul National University, Seoul, Korea; University of Melbourne, Australia

## Abstract

For successful colonization and further reproduction in host plants, pathogens need to overcome the innate defenses of the plant. We demonstrate that a novel pathogenicity gene, *DES1*, in *Magnaporthe oryzae* regulates counter-defenses against host basal resistance. The *DES1* gene was identified by screening for pathogenicity-defective mutants in a T-DNA insertional mutant library. Bioinformatic analysis revealed that this gene encodes a serine-rich protein that has unknown biochemical properties, and its homologs are strictly conserved in filamentous Ascomycetes. Targeted gene deletion of *DES1* had no apparent effect on developmental morphogenesis, including vegetative growth, conidial germination, appressorium formation, and appressorium-mediated penetration. Conidial size of the mutant became smaller than that of the wild type, but the mutant displayed no defects on cell wall integrity. The *Δdes1* mutant was hypersensitive to exogenous oxidative stress and the activity and transcription level of extracellular enzymes including peroxidases and laccases were severely decreased in the mutant. In addition, ferrous ion leakage was observed in the *Δdes1* mutant. In the interaction with a susceptible rice cultivar, rice cells inoculated with the *Δdes1* mutant exhibited strong defense responses accompanied by brown granules in primary infected cells, the accumulation of reactive oxygen species (ROS), the generation of autofluorescent materials, and PR gene induction in neighboring tissues. The *Δdes1* mutant displayed a significant reduction in infectious hyphal extension, which caused a decrease in pathogenicity. Notably, the suppression of ROS generation by treatment with diphenyleneiodonium (DPI), an inhibitor of NADPH oxidases, resulted in a significant reduction in the defense responses in plant tissues challenged with the *Δdes1* mutant. Furthermore, the *Δdes1* mutant recovered its normal infectious growth in DPI-treated plant tissues. These results suggest that *DES1* functions as a novel pathogenicity gene that regulates the activity of fungal proteins, compromising ROS-mediated plant defense.

## Author Summary

Coevolution of plants and microbial pathogens leads to interactions that resemble a molecular war. Pathogens generate effector molecules to infect their hosts, and plants produce defense molecules against pathogen attacks. Interactions between these molecules results in plant immunity or disease. Plant disease could be likened to a complex and delicate matter of balance, where a number of molecules are involved in the battlefield. Discovering and understanding the tipping points in the battle are vital for developing disease-free crops. In the interaction of rice and rice blast fungus, a microbe sensor on rice stimulates the generation of reactive oxygen species (ROS) at the site of infection. ROS is known as an antimicrobial material and a stimulator for defense signaling that is important for preparing reinforcement in neighboring tissues. This paper presents the counter-defense mechanism of the fungus against plant-driven ROS. We found that a pathogenicity factor from rice blast fungus, DES1 (Defense Suppressor 1), is involved in overcoming oxidative stress for the counter-defense mechanism, suggesting that this gene is required for fungal pathogenicity.

## Introduction

Plants are generally immune to most pathogenic microbes due to their innate defense systems, but the exceptional combination of a susceptible host and a pathogen species (or race) can result in disease [1]. Plants have two types of defense mechanism against attack by pathogenic microbes: one against general microorganisms, and the other against specific pathogen races [2],[3]. The general defense mechanism is known as a pathogen-associated molecular pattern (PAMP) triggered immunity (PTI). PTI is initiated by extracellular surface receptors that recognize general features of microorganisms such as bacterial flagellin [4],[5], chitosans (the deacetylated product of chitin [6]), and *N*-acetylchitooligosaccharides (the backbone fragment of the fungal cell wall [7]). As a result of coevolution, plant pathogens have developed various strategies to overcome PTI. One of them is an effector-triggered susceptibility (ETS), which deploys PTI-suppressing pathogen effectors [3]. Many effectors have been identified, and their functions and delivery systems are well studied in Gram-negative bacteria [8]. However, only a few effectors have been reported in plant pathogenic fungi, and their functions in PTI suppression and secretion mechanisms are still unknown [2],[3]. The more specific defense mechanism against pathogen ETS is known as effector-triggered immunity (ETI), which is stimulated by plant surveillance proteins (R-proteins) that specifically recognize one of the pathogen's effector proteins (Avr proteins). ETI is an accelerated and magnified defense response compared to PTI: in bacterial and fungal pathosystems, the same defense genes are related to both defense mechanisms, but they display stronger and faster activation in ETI than in PTI [9],[10]. ETI is accompanied by the active cell death of infected cells, the hypersensitive response (HR), which is known as the ultimate defense mechanism of plants [11]. However, certain pathogens avoid ETI by altering a target effector to prevent the recognition of a particular surveillance protein and/or by deploying other effectors that directly suppress ETI [12],[13].

One of the major and earliest responses of plant PTI is the rapid accumulation of reactive oxygen species (ROS) at the site of infection [14]. ROS act as direct reactive substrates to kill pathogens, to synthesize lignin and other oxidized phenolic compounds that have antimicrobial activity, and to strengthen plant cell walls by oxidative cross-linking to obstruct further extension of the pathogen [15]–[17]. ROS also function as signal molecules for programmed cell death of the infected cell and as diffusible second messengers in the production of various pathogenesis-related (PR) proteins and phytoalexins in neighboring cells [18],[19]. In rice, a membrane OsRac1 GTPase complex, which is required for PTI, controls ROS production through the direct regulation of NADPH oxidase [20],[21]. It is plausible that plant pathogens have counter-defense mechanisms against plant ROS-mediated resistance; however, little is known about how pathogens incapacitate plant-driven ROS. Recently, a study of the AP-1-like transcription factor in the maize pathogen *Ustilago maydis* suggested that peroxidases detoxify host-driven ROS [22].

The Ascomycete *Magnaporthe oryzae*, which causes rice blast disease, is the most destructive pathogen of cultivated rice worldwide [23]. The rice blast pathosystem is a model for studying fungal pathogen–plant interactions not only due to the economic importance of this disease, but also due to the molecular and genetic tractability of both the fungus and the host [24]. Complete genome sequence information is available for both the host and the pathogen, and various molecular functional genomics approaches have been initiated [25]. To investigate pathogenicity genes on a genome scale, our research group has generated >20,000 insertional mutants using *Agrobacterium tumefaciens*-mediated transformation (ATMT) and has evaluated the characteristics of each mutant in the essential steps for disease development [26]. The disease cycle of this pathogen consists of several steps that are essential for successful disease development. Asexual conidia are generated from conidiophores that emerge from diseased lesions and are released into the air. Upon contacting host leaves, conidia become firmly attached by the conidial tip mucilage and germinate upon hydration. Through environmental cues emanating from the plant surface, appressoria, the dome-shaped pre-penetration structures, develop at the end of germ tubes and generate enormous mechanical force to penetrate the outer surface of the plant [27],[28]. After penetration, specialized bulbous biotrophic infectious hyphae (IH) develop before necrotic lesion formation [29],[30]. In this early infectious stage, various interactive reactions are assumed to occur between the fungus and the host, possibly considered as a molecular war, which ultimately determines the level of disease. Therefore, there is growing researches on plant and pathogen factors focusing on this stage [31]. To date, studies on effector proteins in *M. oryzae* have relied on a genetic approach to find avirulence (AVR) genes interacting with plant resistance (R) genes on the early infectious stage. Two avirulence proteins were characterized, *AVR-Pita* [12] and *ACE1* [32], whose putative functions are metalloprotease and polyketide synthase, respectively, but their roles as a virulence factor are insignificant. Study on *MgAPT2*, a member of P-type ATPase, suggested that delivery of fungal effectors including avirulence gene products is essential for infectious growth and HR induction [33]. *Mig1* and *SSD1* are pathogenicity factors dealing with plant innate defense in the early infectious stage, but how they counteract against host defense system and how they contribute to fungal virulence are still unknown [34],[35]. Thus, more detailed study of pathogenicity genes working in the early infectious stage could provide insights into their nature in the plant–fungi interaction.

We identified a T-DNA mutant from the ATMT mutant library, which displayed reduced pathogenicity. Investigation of the mutant led to identification of a fungal-specific gene that is required for plant innate defense suppression: *DES1*. The loss of *DES1* in the fungus leads to the failure of host colonization and induces strong plant defense responses. *DES1* is responsible for compromising oxidative signaling, and its function is related to extracellular peroxidase. Our results suggest that *DES1* serves as a pathogenicity factor that counters plant defenses by restraining the oxidative component of PTI.

## Results

### Identification of a T-DNA mutant with defects in pathogenicity

**Figure 1:**
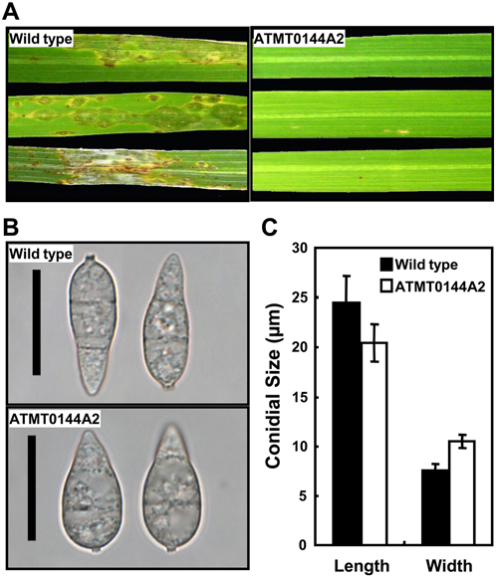
The *Magnaporthe oryzae* T-DNA mutant ATMT0144A2 has defects in lesion development and conidial morphology. (A) Rice seedlings (Nakdongbyeo) were inoculated with the wild-type strain 70-15 (left) and ATMT0144A2 (right). Diseased leaves were harvested 7 days after spray inoculation with conidial suspension (1×10^5^ conidia/ml). (B) Light microscopy of conidia produced by 70-15 (top) and ATMT0144A2 (bottom). Bar = 20 µm. (C) Conidial size of the wild type and ATMT0144A2. Values are the mean±SD from >100 conidia of each strain, which were measured using the Axiovision image analyzer. Length is the distance from the base to apex of conidia. Width is the size of the longest septum.

A T-DNA insertion mutant (ATMT0144A2) showing reduced virulence was identified from the *M. oryzae* ATMT mutant library [26]. This mutant developed restricted resistant-type lesions on a susceptible rice cultivar, Nakdongbyeo, and the number of lesions was much less than in the wild-type strain 70-15 (Fig. 1A). In addition, the mutant produced broader ellipsoidal conidia that were uniform and easily detected under a microscope (Fig. 1B). The conidia of the mutant were on average ∼4 µm shorter and ∼3 µm wider than those of the wild type (Fig. 1C). The T-DNA insertion mutant was not significantly defective in any other mycological phenotype tested, although the mycelial growth rate of ATMT0144A2 was slightly faster than that of the wild type on agar medium (Table 1); the colony morphology of the mutant, however, was indistinguishable from that of the wild type (Fig. S1A). Despite the alteration in conidial morphology, conidia produced by the mutant had no defects in conidial adhesion, germination, and appressorium formation (Table 1). These phenotypes imply that the T-DNA insertion in ATMT0144A2 affects pathogenicity and conidial morphogenesis, but not other pre-penetration developmental stages.

**Table 1:** Comparison of mycological characteristics among strains.

Strain	Mycelial growth^a^ (mm)	Conidiation^b^ (10^5^ per ml)	Conidial adhesion^c^ (%)	Conidial germination^d^ (%)	Appressorium formation^e^ (%)
70-15	63.5±1.0 B	102.3±3.8 B	90.9±5.7 A	100±0.0 A	99.1±1.5 A
DES1^T-DNA^	69.0±1.7 A	108.7±7.6 B	91.1±5.4 A	100±0.0 A	99.0±1.7 A
*Δdes1*	61.3±1.2 B	188.0±5.3 A	94.3±4.0 A	99.7±0.5 A	99.7±0.5 A

Within columns, means with different letters are significantly different, as estimated using Duncan's multiple range test (*P* = 0.05).

a Growth was measured as the diameter of the mycelium 12 days after inoculation.

b Conidiation was assayed by counting the number of conidia from the same culture plates used in growth measurements, flooded with 5 ml of sterile distilled water.

c Conidial adhesion ability was measured as the ratio of attached conidia counted after washing three times in distilled water to total conidia counted before washing.

d Germination ability was measured as the ratio of germinating conidia to total conidia.

e Appressorium formation was measured as the ratio of appressorium-forming conidia to germinating conidia on hydrophobic microscope coverslips.

### ATMT0144A2 phenotypes are caused by a single T-DNA insertion

**Figure 2:**
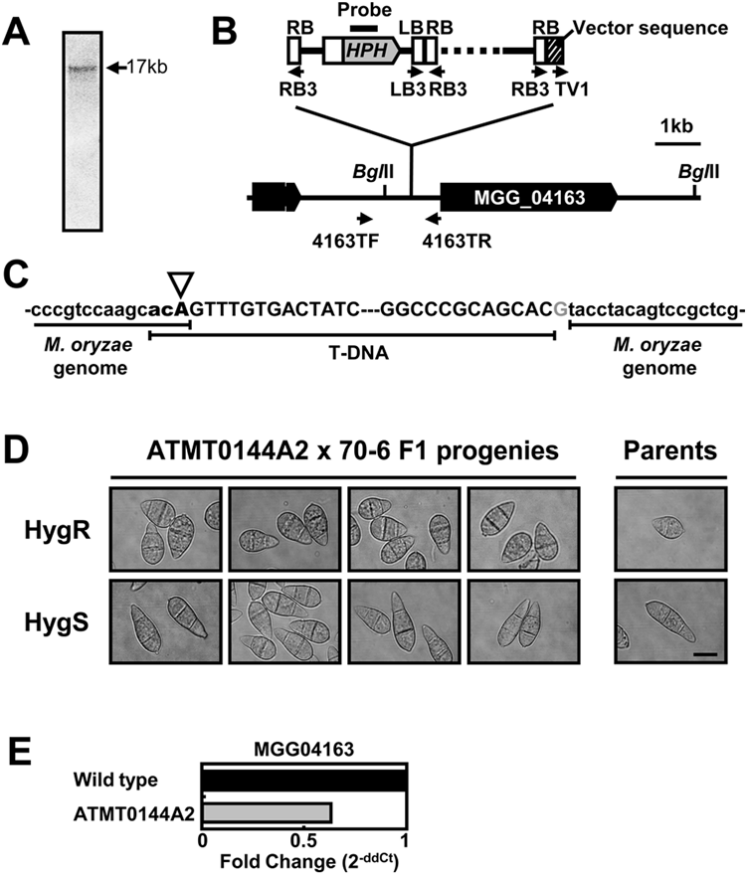
An abnormal T-DNA is integrated in the promoter region of MGG04163. (A) Southern hybridization with ATMT0144A2. Total genomic DNA was digested with *Bgl*II and probed with the *Hpa*I-digested *HPH* fragment. (B) Schematic diagram of T-DNA in ATMT0144A2. Specific primers used for the confirmation of T-DNA insertion (small arrows), vector read-through (slashed box) and unknown regions (dashed line) are indicated. The T-DNA insertion point is –750 from MGG04163 start codon. (C) Sequences of the T-DNA junction sites. Sequences of both junctions between T-DNA (upper case letters) and the *M. oryzae* genome (lower case letters) are indicated. Typical right-border cleavage site (white arrowhead), micro-homology region (bold), and a filler DNA (gray) are denoted. (D) Co-segregation of conidial morphology and T-DNA in F1 progeny. Seven-day-old conidia produced by randomly selected F1 progeny from ATMT0144A2×70-6 crosses were observed under a light microscope, and they were examined on TB3 medium containing 200 ppm hygromycin B. Bar = 10 µm. (E) The transcriptional expression of MGG04163 in ATMT0144A2. The transcription level of MGG04163 was assayed by quantitative RT-PCR using mycelia of the wild type and ATMT0144A2 in 3-day-old liquid culture.

Southern hybridization revealed that ATMT0144A2 has a single insertion of T-DNA in its genome (Fig. 2A). The presence of a single band of ∼17 kb from *Bgl*II-digested DNA suggested the abnormal insertion of several copies of T-DNA at the same locus, since the band location was different from the expected (9 kb). The insertion locus was identified using thermal asymmetric interlaced polymerase chain reaction (TAIL-PCR) [36] with T-DNA border primers, as described in a previous study [37]. The PCR reactions using right border (RB) primers produced two distinct bands (RB-A and RB-B), but the left border (LB) primers produced no detectable band. Sequences from RB-A were matched to the supercontig 6.12 of the *M. oryzae* genome, and sequences from RB-B were matched to the pBHt2 vector region that is adjacent to RB. Both tandem and inverse repeats of T-DNA were detected by amplification with LB and RB primer combinations. From these results, a schematic diagram of the T-DNA integration in ATMT0144A2 was configured, and the insertion pattern was confirmed by PCR amplification with combinations of border primers and locus-specific primers, in which three to four copies of the T-DNA units were tandemly and inversely integrated (Fig. 2B). Junction sequences between the T-DNA and the *M. oryzae* genome revealed that the T-DNA had a typical RB border at one end, but had an abnormal RB read-through and 1-bp filler DNA at the other end (Fig. 2C). As a result of the T-DNA insertion, 6 bp of genomic DNA were deleted from the insertion site. During the in depth study of ATMT0144A2, it was found that the location of the T-DNA insertion was the same as that in another pathogenicity-defective mutant, ATMT0144B3, which also produced broad ellipsoidal conidia [26].

To confirm the single insertion and a correlation between the T-DNA insertion and ATMT0144A2 phenotypes, genetic analyses were performed with two different mating tester strains: 70-6 and 4091-5-8. The integrated T-DNA in ATMT0144A2 was stably segregated to F1 progeny in the genetic crosses. Of 102 F1 progeny from the ATMT0144A2×70-6 cross, 49 progeny were resistant to hygromycin B (HygR), whereas 53 progeny were susceptible (HygS). Chi-square analysis effectively supported 1∶1 segregation (*Χ* ^2^ = 0.09) at the 5% level of significance. All HygR F1 progeny from the cross produced broader ellipsoid conidia like the mutant parent, whereas all HygS F1 progeny produced normal shaped conidia like the wild-type parent (Fig. 2D). PCR amplification between 4163TF and RB3 revealed that all HygR F1 progeny had the T-DNA, whereas all HygS progeny had no T-DNA (data not shown). Thus, the T-DNA insertion is tightly linked to a locus that determines conidial morphology. In the genetic cross ATMT0144A2×4091-5-8, progeny from a single tetrad were the same. The T-DNA was located in a noncoding region between MGG04162.6 and MGG04163.6. MGG04163.6 was the nearest ORF from the T-DNA (750 bp upstream of the start codon), and quantitative RT-PCR showed that the expression level of MGG04163.6 in ATMT0144A2 was reduced to 60% of that in the wild type (Fig. 2E). Therefore, it seems that the integration of the T-DNA at the promoter region of the gene reduced the transcriptional expression and consequently affected pathogenicity and conidial morphogenesis. We named the gene MGG04163.6 (GenBank accession number: XP_361689) as *DES1*, derived from plant defense suppression. Because the T-DNA was present at the 3′-direction of MGG01462.6 beyond the stop codon with a distance of 2.5 kb, it is unlikely that the locus is responsible for the phenotypes of ATMT0144A2. It is further confirmed that the expression level of MGG04162.6 in ATMT0144A2 was not significantly different (1.15 fold) to that of the wild type when examined with quantitative RT-PCR (data not shown).

### *DES1* encodes an unknown fungal-specific protein

The *DES1* gene was located on chromosome IV in the genome of *M. oryzae*, and the predicted ORF was 3,864 bp long, encoding 1,287 amino acids, and there was no intron on the ORF. The sequence of the *DES1* transcript was confirmed by sequencing the cDNA synthesized from mycelial mRNA using four pairs of primers spanning the ORF, and it was identical to the predicted ORF in the *M. oryzae* genome version 6 (data not shown). BLAST searches to find *DES1* homologs resulted in only a few matches to hypothetical proteins of filamentous fungi (Table S1). Additionally, the *DES1* homologs were intensively analyzed in 59 recently released fungal genomes (including three Oomycetes) using the BLASTMatrix tool, which plots the BLAST results by taxonomic distribution (http://cfgp.snu.ac.kr [38]). Interestingly, *DES1* homologs were found only in subphylum Pezizomycotina of Ascomycota, and each homolog was present as a single copy in each genome (Table S1). Of 21 fungal species belonging to Pezizomycotina, the *DES1* homologs were found in 20 species, the exception being *Mycosphaerella graminicola* (Table S1). Sequence alignment of the *DES1* homologs revealed that they are well conserved in length and amino acid composition (Fig. S2), and they were grouped in distinct phylogenetic clades (Fig. S3).

To predict the biochemical function of *DES1*, the amino acid sequences of *DES1* and its homologs were analyzed using the bioinformatics tools InterProScan [39], SignalP [40], and amino acid frequency analysis. Most of the *DES1* homologs had no known functional domain when searched with InterProScan (v12.0). Exceptionally, Afu2g05410, the *DES1* homolog in *Aspergillus fumigatus*, had the IPR002048 (calcium-binding EF-hand) domain consisting of 13 residues in the C-terminal region. Signal P (v3.0) predicted that all of the *DES1* homologs had no signal peptide, indicating that they are likely non-secretory proteins. Amino acid frequency analysis revealed that *DES1* and its homologs are serine-rich proteins: the average serine frequency of *DES1* and its homologs was 13.25%, whereas that of whole *ab initio* annotations of *M. oryzae* was only 7.97%.

### Targeted gene replacement of *DES1* in *M. oryzae*

Targeted gene deletion of *DES1* confirmed that the gene is required for *M. oryzae* lesion development and conidial morphogenesis. A gene deletion vector was constructed by double joint PCR [41], in which the hygromycin resistance gene (*HPH*) cassette was combined with ∼1-kb-long flanking regions (Fig. S4A). The gene deletion vector was introduced to wild-type protoplasts by PEG-mediated fungal transformation. After primary PCR screening of hygromycin-B-resistant transformants using a locus-specific primer (DES1KOSF) and an *HPH* gene primer (HPHF), a *DES1* deletion mutant (*Δdes1*) and an ectopic transformant (E41) were confirmed by Southern hybridization (Fig. S4B). RT-PCR confirmed the null mutation of *DES1* in which the *Δdes1* mutant produced no *DES1* transcript (data not shown). The morphology of conidia produced by the *Δdes1* mutant was similar to that of conidia produced by ATMT0144A2 (designated as DES1^T-DNA^). Conidia produced by the *Δdes1* mutant were significantly shorter in length than those of the wild type, although they did not become as wide as those of DES1^T-DNA^ (Fig. S4C and Fig. S1B). Conidia produced by an ectopic transformant exhibited the normal morphology of the wild type (Fig. S4C). The *Δdes1* mutant was not defective in other mycological phenotypes, including growth rate and colony morphology on CM; conidial adhesion on hydrophobic surfaces; and development of germ tubes and appressorium formation (Table 1).

### The *DES1* gene is required for successful colonization of host tissues

**Figure 3:**
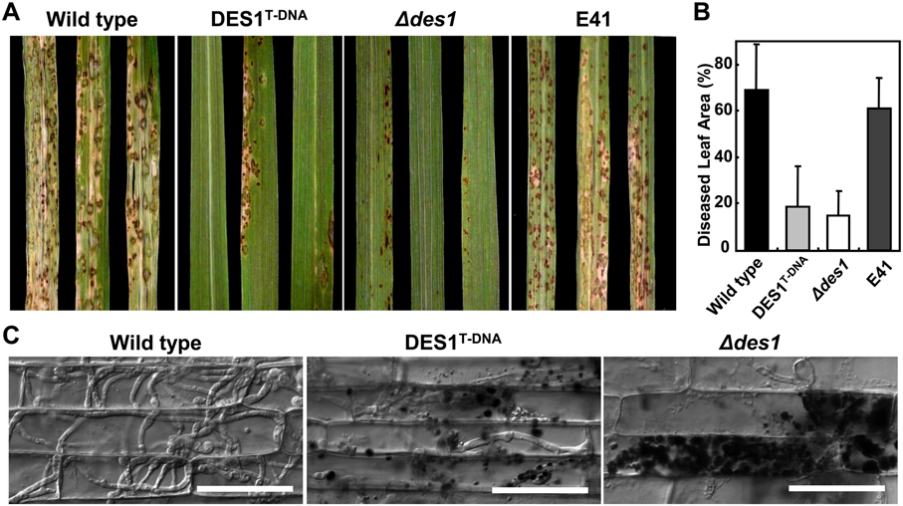
The loss of *DES1* leads to reduced pathogenicity and a colonization defect in host tissues. (A) Pathogenicity assay. Five milliliters of conidial suspension (1×10^5^ conidia/ml) of each strain were sprayed on rice seedlings (Nakdongbyeo). Diseased leaves were harvested 7 days after inoculation. (B) The disease severity of each strain was assessed from the percentage diseased leaf area as calculated using the Axiovision image analyzer. Values are the mean±SD from eight rice leaves inoculated by each strain. (C) Observation of infectious growth. Excised rice sheath from 5-week-old rice seedlings (Nakdongbyeo) was inoculated with conidial suspension (1×10^4^ conidia/ml of each strain). Infectious growth was observed 96 h after inoculation. Bar = 50 µm.

In spray-inoculation tests, the *Δdes1* mutant produced tiny and restricted lesions on a susceptible rice cultivar, Nakdongbyeo, whereas the wild type and the ectopic transformants caused susceptible-type spreading lesions. The level of virulence of DES1^T-DNA^ was intermediate to those of the wild type and *Δdes1* (Fig. 3A). Differences in disease severity were more dramatic when the diseased leaf area (%DLA) was measured. The %DLA of *Δdes1* was 15±10%, which was less than one-quarter that of the wild type (68±20%) and the ectopic transformant (61±13%). The DES1^T-DNA^ showed slightly higher levels of DLA (19±14%) compared to *Δdes1* (Fig. 3B).

**Figure 4:**
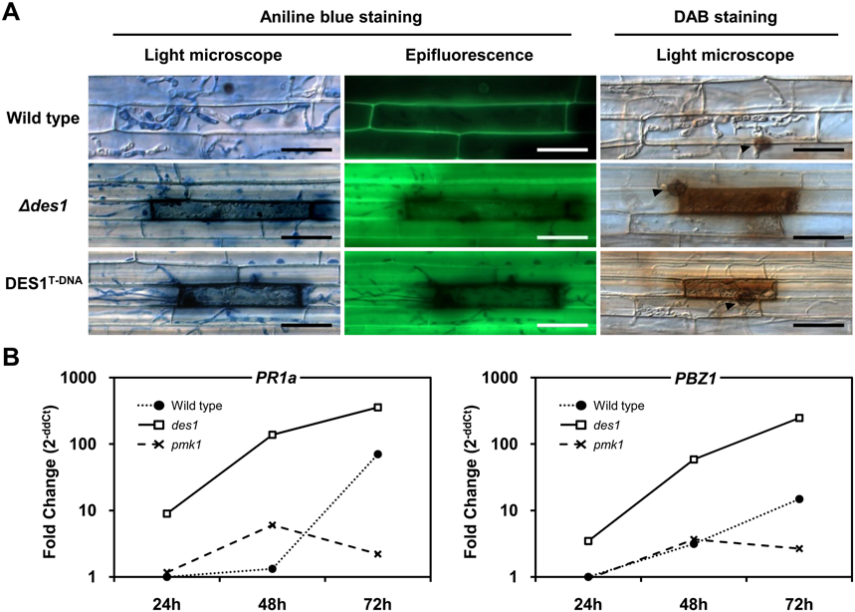
The deletion of *DES1* caused the induction of strong plant defense responses. (A) DIC and fluorescence microscopy of infected rice sheaths (Nakdongbyeo) 48 h after inoculation. DIC images were captured using an 80-ms exposure time of transmission light with a DIC filter. Fluorescence images were captured using a 500-ms exposure for absorbed light using a GFP filter. Arrowheads on DAB staining panel indicate appressorium. Bar = 30 µm. (B) The expression of rice pathogenesis-related (PR) genes over time after inoculation. The transcriptional expression of *PR1a* and *PBZ1* in the infected rice was analyzed using quantitative RT-PCR.

Because no defect in appressorium development was observed in the *Δdes1*mutant, the development of infectious hyphae (IH) within the host cells was examined using an excised leaf sheath assay [42]. IH of the wild-type actively grew and occupied 10–20 cells neighboring the primary infected cells by 96 h after inoculation. However, IH of *Δdes1* were mostly restricted to the primary infected cells, and there was an abundant accumulation of dark brown granules along IH of *Δdes1* (Fig. 3C). Only a few IH of *Δdes1* extended into neighboring cells (Fig. 3C). The DES1^T-DNA^ displayed an intermediate phenotype, with poorly growing IH and scattered dark brown granules along the IH (Fig. 3C). The development of IH was further observed after destaining the brown granules with lactophenol and staining the IH with aniline blue. At 48 h after inoculation, bulbous IH of the wild type filled the primary infected cell, and the IH extended to neighboring cells. Contrary to the wild type, IH of the *Δdes1* and DES1^T-DNA^ mutants seemed to be broken down within primary infected cells, and very few slender IH were present in neighboring cells (Fig. 4A).

### Plant defense responses were induced by challenge with the *Δdes1* mutant

Defense responses induced by the recognition of microbe-associated molecules are often associated with cell wall strengthening, the rapid production of ROS, and the transcriptional activation of PR genes [43]. Because rice cells infected by the *Δdes1* mutant displayed brown granule generation and cell death, it is likely that plant defense responses might be involved in virulence attenuation of *Δdes1*. Thus, the defense responses against the wild type and the mutants were compared.

Autofluorescence at the site of infection indicates the accumulation of phenolic compounds and cell wall strengthening [44]. Under a fluorescence microscope, primary rice cells infected by the wild type emitted autofluorescence only in their cell walls (Fig. 4A). The fluorescence was severely diminished or absent in secondary and further infected rice cells and even in the cells that were occupied by actively growing IH (Fig. 4A). In contrast, strong autofluorescence was observed not only in rice cells directly infected by IH of the *Δdes1* and DES1^T-DNA^ mutants, but also in neighboring cells that were not in contact with the fungus (Fig. 4A).

The accumulation of hydrogen peroxide (H_2_O_2_) at infection sites was also examined by staining with 3,3′-diaminobenzidine (DAB) 48 h after inoculation. Rice cells containing wild-type IH were not stained with DAB, whereas primary infected rice cells with *Δdes1* and DES1^T-DNA^ were strongly stained with DAB, indicating high concentrations of H_2_O_2_ (Fig. 4A). Regardless of the plant responses, appressoria of both the wild type and mutants stained equally with DAB (Fig. 4A, arrowheads in DAB staining). The extent of defense responses seemed to be proportional to the level of *DES1* expression because the levels of autofluorescence and H_2_O_2_ accumulation were lower in cells infected by the DES1^T-DNA^ mutant compared to those infected by the deletion mutant (data not shown).

To further investigate whether the plant defense genes were stimulated by infection with *Δdes1*, the expression patterns of two PR genes were analyzed by quantitative RT-PCR. A MAP-kinase mutant (*pmk1*), which is unable to infect plant tissue [45], was used as a negative control. Upon inoculation with wild-type conidia, the expression of *PR1a* and *PBZ1* followed the typical pattern of compatible interaction, where induction of these genes was delayed to 72 hpi [9]. In contrast, expression of *PR1a* and *PBZ1* was highly induced even in 24 and 48 hpi by inoculation with *Δdes1* (Fig. 4B). Induction levels of *PR1a* and *PBZ1* expression in *Δdes1* challenged rice leaves were 104 and 19 folds at 48 hpi, respectively, compared to those in wild type challenged rice leaves (Fig. 4B). There was a little induction of *PR1a* or *PBZ1* gene expression by inoculation with the *pmk1* mutant, but the induction level was less or not much more than that in wild type challenged rice leaves (Fig. 4B). These results indicate that the induction of plant defense responses in *Δdes1* challenged rice may contribute the retardation of the IH development.

### Inhibition of plant ROS generation restores IH development of the *Δdes1* mutant

**Figure 5:**
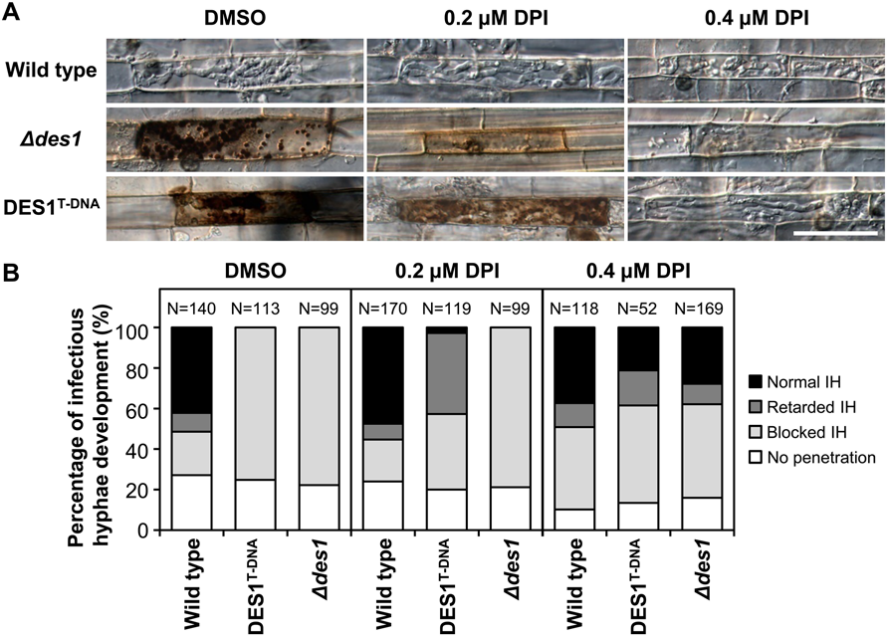
The inhibition of ROS generation recovers the infectious growth of the *Δdes1* mutant. (A) The excised sheath of rice (Nakdongbyeo) was inoculated with conidial suspension (1×10^4^ conidia/ml) of 70-15, *Δdes1*, or DES1^T-DNA^ with or without diphenyleneiodonium (DPI) dissolved in DMSO. Samples were harvested and observed 48 h after inoculation. Bar = 50 µm. (B) Percentage of appressorium-mediated penetration and infectious hyphae development of 70-15, *Δdes1*, and DES1^T-DNA^ in DPI treated onion epidermis. The total number of appressorium is indicated above each column. The level of IH development were scored after 72 h after inoculation (see Materials and Methods for details).

Plant NADPH oxidases generate ROS in response to pathogen attack [19]. To determine whether the virulence of the *Δdes1* and DES1^T-DNA^ mutants is affected by ROS generation in host plant tissues, diphenyleneiodonium (DPI), an inhibitor of NADPH oxidases [46], was applied to the rice sheath. Since treatment of high concentration (>25 µM) of DPI could affect conidial germination [47], we used 0.2–0.4 µM of DPI to prevent the effects on the fungal development. At these concentrations, conidial germination, appressorium-mediated penetration and IH development were not affected in the wild type (Fig. 5A). However, the attenuated virulence phenotypes of *Δdes1* and DES1^T-DNA^ were rescued in the rice sheath cells in which ROS generation was inhibited by DPI. Treatment with 0.2 µM DPI resulted in the reduction and fragmentation of the dark-brown granules around IH in the mutants (Fig. 5A). IH of the *Δdes1* mutant were still restricted in the presence of 0.2 µM DPI, but IH of DES1^T-DNA^ successfully occupied the primary infected cell and extended to neighboring cells (Fig. 5A). Treatment with 0.4 µM DPI completely prevented the production of brown granules, and both mutants could develop IH (Fig. 5A).

The level of plant defense response and the IH development were measured on onion epidermis. Under normal conditions (without DPI), ∼70% of the appressoria of the wild type penetrated into onion epidermis, but finally ∼40% of the appressoria successfully developed IH due to plant defense responses including callose deposition (Fig. 5B and Fig. S5). In contrast, only a few penetrated appressoria of *Δdes1* and DES1^T-DNA^ developed IH, although the penetration rate was similar to that of the wild type (Fig. 5B and Fig. S5). Treatment of DPI (0.4 µM) recovered the frequencies of IH development by *Δdes1* and DES1^T-DNA^ up to 28% and 21%, respectively (Fig. 5B and Fig. S5). The shapes of the recovered IH of *Δdes1* and DES1^T-DNA^ were not distinguishable from that of the wild type (Fig. 5A and Fig. S5). Similar to the results of the rice sheath test, the level of defense response of onion seemed to be related to DPI concentration and the expression level of the *DES1* gene. These results suggest that the *DES1* gene is related to either suppression of defense initiation in rice and onion (by similar mechanisms of DPI) or overcoming the defense responses by degrading brown granules and callose. Re-introduction of wild-type allele of *DES1* gene into the *Δdes1* mutant also recovers IH development in rice sheath and onion epidermis and the ability to suppress the plant basal defense (Fig. S6). This result indicates that the deletion of *DES1* gene is the very reason for the failure of infectious growth and PTI suppression.

### The *Δdes1* mutant is hypersensitive to oxidative stress

**Figure 6:**
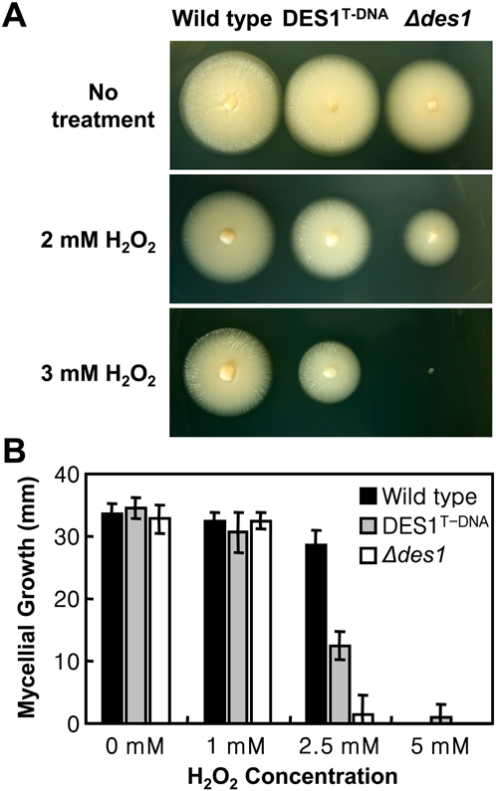
The *Δdes1* mutant is hypersensitive to oxidative stress. (A) Mycelial colonies on complete agar medium with or without 2–3 mM H_2_O_2_ on day 4 after inoculation. (B) Mycelial growth on complete medium with or without H_2_O_2_ (1–5 mM) on day 7 after inoculation. The colony diameters of four replicates were measured. Error bars represent the standard deviation.

Because DPI is known to suppress plant ROS production, we investigated whether the *DES1* gene is required to modulate either ROS or other diverse stress conditions that fungal pathogens may encounter in the plant cells. The *Δdes1* and DES1^T-DNA^ mutants did not show any differences in mycelial growth with high concentrations of osmolytes such as 1 M sorbitol or 0.5 M NaCl (data not shown). However, the mycelial growth of *Δdes1*and DES1^T-DNA^ was severely affected under oxidative stress conditions (Fig. 6A). The growth of mutants was altered at 2–3 mM H_2_O_2_, and the level of sensitivity was more significant in *Δdes1* than in DES1^T-DNA^. The growth of the wild type was not significantly affected under these same conditions (Fig. 6B). These results indicate that the *DES1* gene is related to ROS degradation.

**Figure 7:**
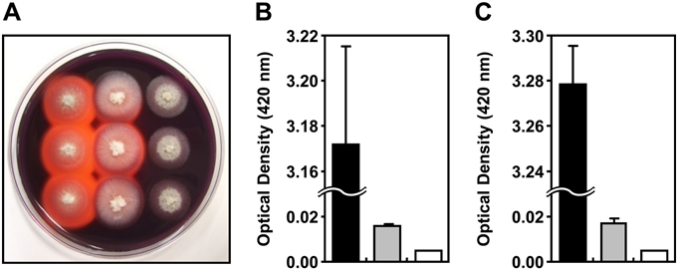
*DES1* is related to activity of extracellular peroxidase and laccase. (A) The discoloration of Congo Red was tested on medium containing 100 ppm of the dye at final concentration. Strains were inoculated on CM agar medium containing Congo Red. Discoloration was observed on day 4. Left: wild type, middle: DES1^T-DNA^, right: *Δdes1*. (B) Peroxidase activity measured by ABTS oxidizing test under H_2_O_2_ supplemented condition (see Materials and Methods for details). Black column: wild type, grey column: DES1^T-DNA^, white column: *Δdes1*. (C) Laccase activity measured by ABTS oxidizing test without H_2_O_2_. The strain scheme is same with panel B. Error bars represent standard deviation.

Since the conidial morphology of *Δdes1*and DES1^T-DNA^ was altered, hypersensitivity to oxidative stress and reduced virulence may also be due to defects in cell wall composition, in spite of their insensitivities to osmotic stresses. To investigate this possibility, we added Nikkomycin Z, a chitin synthetase inhibitor, to germinating conidia. Treatment of Nikkomycin Z blocks conidial germination and induces protoplast-like swellings on cell wall-defective strains [48]. However, conidial germination of *Δdes1*and DES1^T-DNA^ was not inhibited in high concentrations of Nikkomycin Z (100 µM) and swellings on germ tubes were not distinguishable from the wild type (Fig. S7A). We also tested the sensitivity of these strains to a cell wall-degrading enzyme. Enzyme-treated mycelia of *Δdes1*and DES1^T-DNA^ released no more or less protoplasts than the wild type when observed over a time course (Fig. S7B). We also tested mycelial growth on Calcofluor white (CFW) and Congo Red (CR) amended media, which inhibit fungal cell wall assembly by binding chitin and β-1,4-glucans, respectively [49],[50]. The mycelial growth of *Δdes1*on CFW media (100 ppm) was little reduced (88% of the wild type) when compared with normal CM (96% of the wild type), and it was more severely reduced (70% of the wild type) on CR media (100 ppm). However, since degradation halo was observed around the wild-type colony and no degradation halo was observed around the *Δdes1* colony (Fig. 7A), the growth defect on CR media was assumed to be due to the absence of CR-degrading activity rather than defects in cell wall composition. The DES1^T-DNA^ colonies showed intermediated levels of CR discoloration (Fig. 7A).

### The *DES1* gene is related to the activity of extracellular peroxidase

Since discolored halos were observed beyond the colony margins, extracellular enzymes were presumed to be responsible for CR degradation. Because the CR degradation reaction is known to be catalyzed by peroxidase, which requires H_2_O_2_ as a limiting substance [51],[52], we tested the influence of H_2_O_2_ on CR discoloration in *M*. *oryzae*. The sizes of the discolored halos increased considerably around the wild type when 1 mM H_2_O_2_ was added to CR medium. No color change was observed with the *Δdes1* mutant, regardless of H_2_O_2_ treatment (data not shown). Considering the defective phenotypes of *Δdes1* in scavenging H_2_O_2_ and discoloring CR, we reasoned that the *DES1* gene is involved in extracellular peroxidase activity. Enzyme activity assay using 2, 2′-azino-di-3-ethylbenzthiazoline-6-sulphonate (ABTS, Sigma, A1888) [53] as substrate revealed that the *Δdes1* mutant totally lost its peroxidase activity in extracellular culture filtrate (Fig. 7B). The culture filtrate of DES1^T-DNA^ showed very low level of peroxidase activity (Fig. 7B). In addition, the ABTS oxidation test without H_2_O_2_ revealed that laccase activity [54],[55] was also diminished in the culture filtrates of *Δdes1*and DES1^T-DNA^ (Fig. 7C). We also compared the activity of another extracellular enzyme, xylosidase, in the culture filtrate of *Δdes1*, DES1^T-DNA^, and the wild type. However, xylosidase activities of the mutant strains were not different from that of the wild type (data not shown).

### Deletion of *DES1* affects the expression of several groups of peroxidase genes

**Figure 8:**
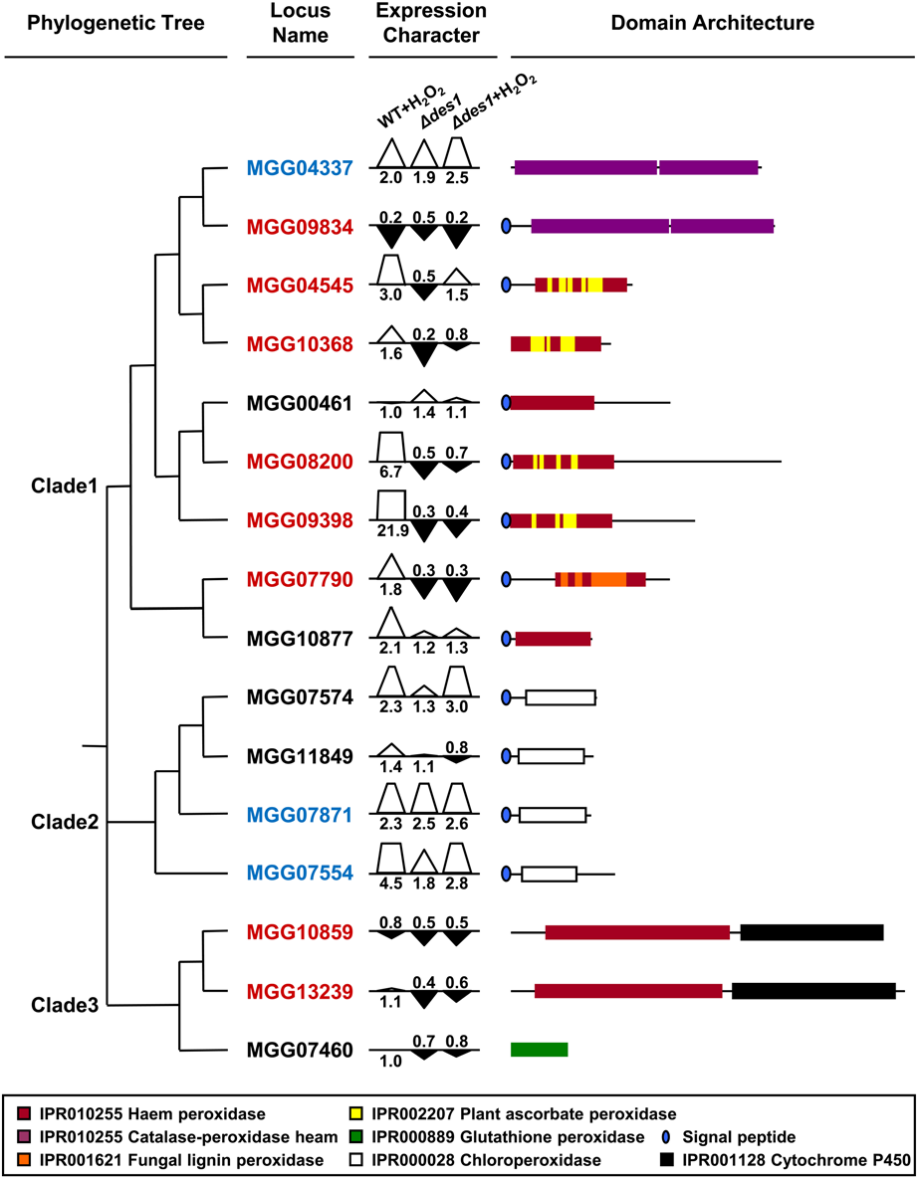
Expression Profiles of *M. oryzae* Peroxidases in the *Δdes1* Mutant. A combination of the phylogenetic tree, expression characteristics, and domain architecture of 16 putative peroxidases in the *M. oryzae* genome were displayed. The phylogenetic tree was generated by ClustalW sequence alignment with 1000 bootstrappings and divided into three clades. The transcript levels of the the putative peroxidase encoding genes in the oxidative condition and/or in the *Δdes1* mutant are indicated. Relative abundance of transcript compared with standard condition (wild type, normal condition) is displayed as a white triangle (up-regulated) or an inverted black triangle (down-regulated). Triangles indicating more than 2.0 (fold change) are displayed as trapezoids by cutting the top of the triangle. Fold changes of the standard condition (1.0) are not shown. Up-regulated genes in the *Δdes1* mutant (more than 1.5 fold) were indicated in blue, and down-regulated genes in the *Δdes1* mutant (less than 0.6 fold) were indicated in red. The InterPro terms and signal peptides are indicated (see legend).

We examined the transcriptional regulation of genes encoding peroxidases. Putative peroxidase-encoding genes were identified from the annotated *M. oryzae* genome database. There were 19 such genes that had peroxidase-related InterPro domains, including IPR010255 (haem peroxidase), IPR000889 (glutathione peroxidase), and IPR000028 (chloroperoxidase). Three of these 19 genes were excluded because their transcripts were not detected under the given experimental conditions. Sixteen putative peroxidase genes in *M. oryzae* could be classified into three clades by phylogenetic analysis, and most of them possessed a signal peptide when assessed using the SignalP program (Fig. 8). Differences in the transcriptional expression of the peroxidase genes between the wild type and the *Δdes1* mutant under oxidative (1 mM H_2_O_2_) or normal (no H_2_O_2_) conditions were examined using quantitative RT-PCR, and fold changes were calculated using wild type under normal condition as a standard condition. The expression level of some peroxidase genes in clade 1 (plant ascorbate peroxidases: MGG04545, MGG10368, MGG08200, and MGG09398; fungal lignin peroxidase: MGG07790) was up-regulated under the oxidative condition in the wild type (Fig. 8). The transcription of MGG07790, MGG08200, and MGG09398 was down-regulated in the *Δdes1* mutant, and the reduced transcription was not recovered by treatment with H_2_O_2_. The expression of MGG10368 and MGG04545 was also repressed in the *Δdes1* mutant, but the reduced transcription was partially recovered by treatment of H_2_O_2_ (Fig. 8). The expression level of the other peroxidase genes in clade 1 (catalase peroxidases: MGG04337 and MGG09834; haem peroxidases: MGGG00461 and MGG10877) was not significantly altered by the deletion of the *DES1* gene. Peroxidase genes in clade 2 (chloroperoxidases: MGG07574, MGG11849, MGG07871, and MGG07574) were responsive to H_2_O_2_ treatment, but their expression was not down-regulated in the *Δdes1* mutant. Peroxidase genes in clade 3 (cytochrome P450 peroxidases: MGG10859 and MGG13239; glutathione peroxidase: MGG07460) were not responsive to H_2_O_2_ under the experimental conditions, but their expression was down-regulated in *Δdes1* (Fig. 8). The expression of putative laccase-encoding genes was also examined in the *Δdes1* mutant. Seventeen genes having two or three multicopper oxidase domains (IPR001117, IPR011706, or IPR011707) were identified from the *M. oryzae* genome database. We excluded one of them (MGG09102) from the analysis because the transcript was not detected in the given experimental conditions. Expression levels of laccase genes in the oxidative condition (1 mM H_2_O_2_) were rather reduced or similar in wild type. Transcription of all laccase genes except one (MGG07500) was also down-regulated in the *Δdes1* mutant, even differences in the transcription level of the laccase genes between wild type and the *Δdes1* mutant were more severe than those of the peroxidase genes (Fig. S8).

### The *DES1* gene is required for regulation of ferrous ions

Since the DES1 gene has no DNA-binding domain, assuming that *DES1* acts as a direct transcriptional regulator of genes encoding peroxidase and laccase was difficult. So we investigated whether the *Δdes1* mutant has defects in metal ion regulation, which may affect the expression and activity of enzymes with a metal core, including peroxidase and laccase. To investigate this hypothesis, bathophenanthroline sulfonate (BPS, Sigma, B1375), a chromogenic, and a specific chelator of the ferrous ion were used to detect extracellular ferrous ions [56]. Since complete media (CM) includes ∼25 µM of ferrous ions, we used CM without trace elements as a negative reference for spectrophotometry. The BPS color reaction was stronger in the *Δdes1* culture filtrate than in the wild type culture filtrate, and of an intermediate color in the DES1^T-DNA^ culture filtrate (Fig. S9). This result suggests that the DES1 is related to either uptake or storage of ferrous ions.

### Subcellular localization of the DES1 protein

**Figure 9:**
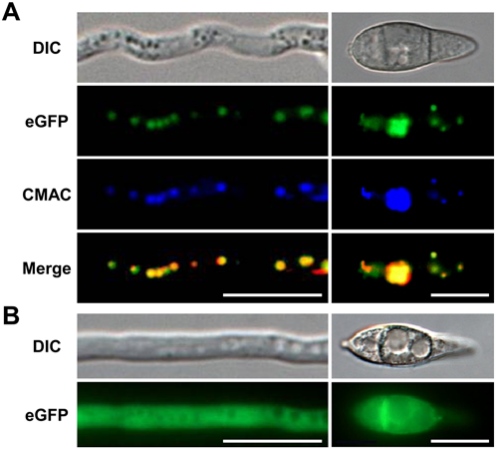
DES1-eGFP is localized in vacuoles. (A) Growing hyphae (left) and conidia (right) expressing DES1-eGFP in Czapek-Dox media. In the merged image, the original blue color from CMAC was changed to red for better visualization, so the co-localized spots were indicated as yellow. Bars = 10 µm. (B) Growing hyphae (left) and conidia (right) expressing eGFP without DES1 in Czapek-Dox media. Bars = 10 µm.

To identify the cellular component to which the DES1 protein is targeted, a fluorescent reporter gene (eGFP) was fused to the C terminus of the *DES1* gene. Since GFP observations using the native promoter (∼1.2 kb) were not successful (data not shown), we used the *Aspergillus nidulans TrpC* promoter for constitutive expression. The Pro*_TrpC_*-*DES1*-eGFP fusion construct and a plasmid containing the geneticin resistance gene (pII99) were introduced into wild-type protoplasts by co-transformation. DES1-eGFP fusion proteins were targeted to vacuoles in the conidia and growing mycelia of these transformants whereas eGFP without DES1 protein, which was expressed by the same *TrpC* promoter, was distributed to the cytosol (Fig. 9). Co-localization of the fluorescence signals with the vacuole-indicating dye 7-amino-4-chloromethylcoumarin (CMAC) confirmed the vacuolar localization of DES1-eGFP fusion proteins (Fig. 9).

### Expression profiles of the *DES1* gene

The transcriptional expression of *DES1* during fungal development was analyzed using quantitative RT-PCR. The cyclophilin-encoding gene (*CYP1*, MGG10447), which displayed stable expression during the developmental stages (Kim et al., unpublished data), was used as an endogenous control gene for normalization. Transcripts of the *DES1* gene increased more than 2 folds during conidiation and infectious growth *in planta*. The level of expression did not change significantly under conditions that included conidial germination and appressorium formation (Fig. S10). The expression of *DES1* was 2.8-fold up-regulated by treatment with 1 mM H_2_O_2_ (Fig. S10).

## Discussion

We described a novel pathogenicity gene of *M. oryzae* that we named *DES1*, which plays an essential role in colonization *in planta*. *DES1* (involved in defense suppression of innate plant immunity) was originally identified as a pathogenicity-defective mutant generated by random insertional T-DNA mutagenesis of *M. oryzae* [26]. Subsequently, a gene deletion mutant was generated, and the inoculation of the null mutant on a susceptible rice cultivar resulted in a more significant reduction of pathogenicity than the T-DNA insertion mutant. Strong defense responses were observed along the infectious hyphae of both *Δdes1* and DES1^T-DNA^, and the mutants had no apparent defects in hyphal growth, conidial germination, conidial adhesion, appressorium formation, appressorium-mediated penetration, and cell wall integrity. Moreover, treatment with a plant defense inhibitory chemical (DPI) recovered the IH development of *Δdes1* and DES1^T-DNA^. The reduction in pathogenicity of the mutants seems to be due to strong plant defense responses, which resulted from failure of proper interactions between the host and the pathogen, rather than defects on IH development. Observation of *PR1a* and *PBZ1* inductions in *Δdes1*-challenged rice tissue supports this hypothesis. The defense response against *Δdes1* and DES1^T-DNA^ seems to be stronger than the typical rice PTI, which has been reported as whole-plant specific resistance (WPSR) [42],[57]. However, it does not seem to be the ETI response, which is induced by an AVR–R gene interaction, since it occurred on both rice and onion—genetically different two species. Therefore, we assigned the plant response against *Δdes1*and DES1^T-DNA^ as an intensified or non-suppressed version of PTI.

PTI in rice is triggered by cell wall derivatives of the fungus [58]. It is initiated via a signaling complex that includes a small GTPase OsRac1, which directly activates NADPH oxidases [20],[21],[59]. H_2_O_2_ produced by the NADPH oxidases is essential for the PTI response in plants, not only as a direct antimicrobial material but also a diffusible second messenger for defense gene induction [43]. It is presumed that virulent pathogens have developed ROS scavenging mechanisms to suppress the PTI defense responses of their hosts. The wild type *M. oryzae* has the ability to detoxify ∼2 mM of H_2_O_2_ without disturbing hyphal growth. Since *Δdes1*and DES1^T-DNA^ showed growth defects under the same conditions, it is assumed that the H_2_O_2_-degrading ability in the mutants was lost or severely weakened. Note that the plant defense responses against *Δdes1*and DES1^T-DNA^ seemed to be regulated by the ROS level because not only did treatment with a ROS inhibitor (DPI) resulted in a dramatic reduction of the plant defense response on both rice and onion tissues but also the degree of the defense response was quantitatively affected by both the DPI and *DES1* expression level. Therefore, we hypothesized that ROS scavenging ability controlled by *DES1* is essential to the suppression of oxidative signaling, which is important in the induction of PTI. However, we cannot completely exclude other possibilities that unknown factors may be affected by the deletion of *DES1*.

We showed that the *Δdes1* mutant lost H_2_O_2_-degrading ability. H_2_O_2_ is known to be scavenged by catalase and various peroxidases, including ascorbate and glutathione peroxidases [60]. In the *Δdes1* mutant, the expression of putative peroxidase genes was down-regulated, and CR discoloration, which is known to be catalyzed by peroxidases, was completely abolished. It is thus suggested that some of the secreted peroxidase might be involved in extracellular H_2_O_2_ detoxification in *M. oryzae*. Molina and Kahmann [22] also reported that a transcription factor, *YAP1*, controls the expression of peroxidase genes (*um01947* and *um10672*) in the maize pathogen *U. maydis* and is responsible for the scavenging of host-derived ROS in the fungus–plant interaction. We also found that the expression of a gene homologous to *um01947* (MGG10368) was down-regulated in the *Δdes1* mutant. This suggests that fungal peroxidases might play a role as common PTI suppressing effectors in rice and maize pathogens, although the regulatory mechanism is not likely to be identical (a *DES1* gene homolog is absent in *U. maydis*). The matching homologous gene to *um10672* was not found in the *M. oryzae* genome database. Catalase is also known to scavenge H_2_O_2_ [60]. However, *CATB* in *M. oryzae* seemed to be required for the strengthening of fungal cell walls, rather than the scavenging of host-driven H_2_O_2_ [54], and the expression of *CATA* and *CATB* was not affected by deletion of the *DES1* gene. Therefore, we suggest that the extracellular peroxidases are a more likely candidate for host-driven H_2_O_2_ in rice–*M. oryzae* interactions.

The *Δdes1* mutant had higher levels of ferrous ions in culture media than the wild type, which suggests defects in either uptake or storage of ferrous ions. Furthermore, we found that the DES1 protein is targeted to the vacuole. Since fungal vacuoles are considered to be areas for the storage of metal ions and the regulation of their homeostasis in the cell [61],[62], these results may provide a possible explanation that the *DES1* may function on metal ion homeostasis, which could affect the activity of enzymes with a metal core [63],[64]. Considering that both peroxidase and laccase need a metal cofactor (iron and copper, respectively) and that the activity of laccase in *M. oryzae* is inhibited under copper-depleted conditions [65], these could explain why both activities of peroxidase and laccase were affected by the deletion of *DES1*. Since the *Δdes1* mutant showed defects in regulation of ferrous ions, the DES1 function might be related to that of siderophores, which are high-affinity iron chelators used for iron uptake and storage in many fungal species [66]. In the recent study of a ferrichrome-type siderophore synthetase (SSM1) [67], however, expression of *SSM1* was not correlated to the level of FeCl_3_ concentration, and the *Δssm1* mutant was not sensitive to oxidative stress, unlike the *Δdes1* mutant. These results suggest that there is no direct connection between DES1 and SSM1 in ferrous ion regulation in this fungus. An alteration of intracellular ROS has also been reported to affect appressorium-mediated penetration by *M*. *oryzae* [47],[68]. However, the cellular effects of ROS regulation by *DES1* seem to be limited when compared to that by *ABC3* (a multidrug resistance transporter gene) [68]. Although the *Δdes1* mutant displayed high sensitivity to oxidative stress, unlike *abc3Δ*, *Δdes1* was not lethal on 2 mM H_2_O_2_ and the appressoria of *Δdes1* were fully functional without any treatment with antioxidants. Furthermore, the *Δdes1* mutants generated the same level of DAB-positive material in their appressoria as the wild type. These indicate that deletion of *DES1* does not lead to alteration of intracellular ROS, which are generated by the NADPH oxidases of *M. oryzae* [47],[68]. These observations suggest that *DES1* might be related to the regulation of a limited range of ROS, or only extracellular ROS.

In conclusion, we identified and characterized a novel pathogenicity gene that is required for host colonization using two independent mutants with different alleles: DES1^T-DNA^ and *Δdes1*. *DES1* is responsible for scavenging extracellular ROS within host cells, which in turn results in a counter-defense of the pathogen against the plant innate defense responses. The discovery and functional assignment of more pathogenicity factors affecting plant defense systems may help to understand the nature of plant disease.

## Materials and Methods

### Fungal strains and culture conditions

*Magnaporthe oryzae* strain 70-15 (*Mat1-1*) and 70-6 (*Mat1-2*) were obtained from A.H. Ellingboe (University of Wisconsin-Madison, USA), and 70-15 was used as wild-type strain in this study. Strain 4091-5-8 (*Mat1-2*) was obtained from B. Valent (Kansas State University, USA). All fungal strains are stored in the Center for Fungal Genetic Resources (Seoul National University, Seoul, Korea; http://genebank.snu.ac.kr). Strains were normally maintained on oatmeal agar medium (OMA, 5% oatmeal and 2.5% agar powder (w/v)) and grown at 25°C under constant fluorescent light to promote conidiation. The strains were cultured for 3 to 12 days on complete agar media [69] to assess the growth and colony characteristics. Hygromycin B resistant transformants generated by fungal transformation were selected on solid TB3 agar media (0.3% yeast extract, 0.3% casamino acids, 1% glucose, 20% sucrose (w/v), and 0.8% agar powder) supplemented with 200 ppm hygromycin B. Mycelia used for nucleic acid extraction were prepared by growing the relevant strains in liquid CM for 3 days at 25°C with agitation (150 rpm), or directly obtained from the TB3 agar media for the quick DNA extraction method described previously [70]. Genetic crosses and progeny analysis (tetrads or random ascospore analysis) were performed as previously described [71]. To observe the vegetative growth or RNA extraction under stress conditions, the fungal strains were treated as follows; Oxidative stress was applied by amending solid or liquid CM with the proper volume of H_2_O_2_ solution (Aldrich, 323381, 3 wt. %). Three-day-old mycelia in liquid CM were treated with or without 1 mM of H_2_O_2_ for 30 min before harvesting for RNA extraction. Stress conditions for cell wall biogenesis was performed by supplementation of Congo Red (CR, Aldrich, 860956) and Calcofluor White (CW, Sigma, F3543) in 100 ppm final concentration in CM agar media, both of which are known to interfere with the assembly of fungal cell walls [72]. For the osmotic stress conditions, CM agar media was amended with 500 mM NaCl and 1 M sorbitol in final concentration.

### Developmental phenotypes assays

Radial colony growth rate was measured on CM agar plates 12 days after inoculation with triplicate. Colony color and morphology were also observed in the condition above. Conidiation was assayed with the 12-day-old colonies grown on OMA. Conidia were collected in 5 ml of distilled water by scraping and counted with a haemacytometer under a microscope. Conidial germination and appressoria formation were measured on hydrophobic microscope coverslip (Marienfeld, Landa-Königshofen, Germany). Conidia harvested from 12-day-old OMA culture were diluted into 2×10^4^ conidia per milliliter in sterile distilled water. Drops of conidial suspension (40 µl) were placed on the coverslips with three replicates, then placed in a moistened box and incubated at 25°C. After 8 hr incubation, the percentage of conidial germination and appressorium formation was determined by microscopic examination of at least 100 conidia per replicate in at least three independent experiments.

### Fluorescence microcopy

Fluorescence and DIC imaging was done using a Zeiss Axio Imager A1 fluorescence microscope (Carl Zeiss, Oberkochen, Germany). A filter set with excitation at 470/40 nm and emission at 525/50 nm was used for enhanced green fluorescence protein (eGFP) observation, another filter set with excitation at 365 nm and emission at 445/50 nm was used for 7-amino-4-chloromethylcoumarin (CellTracker™ Blue CMAC, Invitrogen, Carlsbad, CA, USA) and Aniline blue fluorochrome observation. The staining of conidia and growing mycelia with CMAC was performed as previously described [73].

### Pathogenicity assays and infectious growth visualization

For spray inoculation, conidial suspension (10 ml) containing Tween 20 (250 ppm) and conidia harvested from 12-day-old cultures on OMA plate (1–5×10^5^ conidia/ml) was sprayed onto four-weeks old susceptible rice seedlings (*Oryza sativa* cv. Nakdongbyeo). Inoculated plants were placed in a dew chamber at 25°C for 24 hours in the dark, and then transferred back to the growth chamber with a photoperiod of 16 hours using fluorescent lights [74]. Disease severity was assessed at seven days after inoculation. The %DLA was recorded to permit more accurate evaluation of the virulence of the mutants. Photographs of diseased rice leaves including eight centimeter long leaf blades were taken. The number of pixels under lesion areas and healthy areas of diseased leaves was calculated by Axiovision image analyzer with the photographs. For microscopic observation of penetration and infectious growth on rice tissue, excised rice leaf sheath of Nakdongbyeo were prepared as previously described [30],[42] and inoculated by conidia suspension (1×10^4^ conidia/ml) on the adaxial surface. After 24, 48 and 96 hours incubation in a moistened box, the sheaths were trimmed to remove chlorophyll enriched plant parts. Remaining epidermal layer of mid vein (three to four cell layers thick) were utilized for microscopic observations. Inoculation on onion epidermis was performed as previously described [75]. Fixation and aniline blue staining of rice sheath and onion epidermis were performed as previously described [75]. Samples were incubated in lactophenol at room temperature for 1hour and directly mounted with 70% glycerin or transferred into 0.01% aniline blue for 1hour and destained with lactophenol. For 3, 3′-diaminobenzidine (DAB, Sigma, D-8001) staining, samples were incubated in 1mg/ml DAB solution (pH 3.8) at room temperature for 8 hours and destained with clearing solution (ethanol∶acetic acid = 94∶4, v/v) for 1 hour. For observation and scoring penetration rate and IH development, conidia suspension were dropped on onion epidermis and incubated for 72 hours in moistened culture plate. Samples were fixed and stained as rice sheath described above. Extensive IH from single appressoria with no (or scatterd) callose were scored as normal IH, relative short and attenuated IH with accumulated callose were scored as retarded IH, appressorium developing very short IH or penetration peg with strong callose were scored as blocked IH, and appressorium without IH and callose deposition were scored no penetration.

### Nucleic acid manipulation and polymerase chain reaction

For Southern hybridization analysis, genomic DNA was isolated according to the method described [76] with slight modification. Restriction enzyme digestion, agarose gel separation, and cloning were performed following standard procedures [77]. Southern hybridization analysis was carried out as described previously [75]. *HpaI* fragment (1.4 kb) including hygromycin B phosphotransferase gene (*HPH*), and pCB1004 were used as the hybridization probe. Genomic DNA of transformants for PCR screening was isolated by the quick extraction procedure [70]. About 50 ng of genomic DNA (2 µl) was used for PCR reactions with 1µl of 100 nM of each primer and 5 ul of 2× PCR mixture containing dNTP, PCR buffer, 1 unit of *Taq* polymerase and loading dye (Enzynomics™, Daejeon, Korea). Primers used in this study are listed in Table S2. Perkin-Elmer 9600 DNA Thermal Cycler was employed for PCR. Plasmid DNA was prepared by standard methods [77]. Total RNA was isolated from the frozen fungal and plant tissues with Easy-spin™ total RNA extraction kit (iNtRON Biotechnology, Seoul, Korea) according to the manufacturer's instruction. To quantify levels of transcript, quantitative RT-PCR was performed as described [78]. Briefly, 5 µg of total RNA was reverse transcribed into first-strand cDNA with oligo (dT) primer using SuperScript™ First-Strand Synthesis System (Invitrogen™ Life Technologies, Carlsbad, CA, USA) according to the manufacturer's instruction. Reactions were performed in a 25 µl volume containing 100 nM of each primer, 2 µl of cDNA (25 ng of input RNA) and 12.5 µl of 2× Power SYBR® Green PCR Master Mix (Applied Biosystems, Warrington, UK). Real-time PCR was run on the Applied Biosystems 7500 Real Time PCR System (Applied Biosystems, Foster City, CA). After each run, amplification specificity was checked with a dissociation curve acquired by heating the samples from 60 to 95°C. Normalization and comparison of mean Ct values were performed as described [79]. To compare relative abundance of transcripts of target genes, the mean threshold cycle (Ct) of triplicate reactions was normalized by that of *M. oryzae* cyclophilin gene (*CYP1*, MGG10447), which displayed stable expressions in the developmental stages (unpublished data) and used previously [78],[80],[81], or by that of *O. sativa* elongation factor 1α gene (Os03g08020, [82]). Fold changes were compared among treatments or conditions with standard condition. Quantitative RT-PCR was conducted at least twice with three replicates from independent biological experiments. Genomic DNA adjacent to the T-DNA insertion of DES1^T-DNA^ was isolated by TAIL-PCR, which was performed as described previously [37]. The insertion was confirmed by PCR amplification with pairs of locus specific primers (4163TF and 4163 TR, see primer list), and T-DNA specific primers (RB3 and TV1). The amplified PCR fragments were sequenced twice to analyze insertion characteristics.

### Targeted disruption and complementation of *DES1* in *M. oryzae*

The targeted gene disruption vector was designed using modified double-joint PCR [41]. The target region was a ∼4.5 kb size fragment including *DES1* ORF and short putative UTR (5′-70 bp and 3′-500 bp) sequences. An 1188 bp long 5′ flanking region was amplified with a primer pair, DES1KOSF and DES1KO5R. A 942 bp long 3′ flanking region was amplified with a primer pair, DES1KO3f and DES1KOSR. Both 5′ and 3′ flanking regions of target sequences were fused to *HPH* cassette using a specific primer pair with 23 bp tail sequence in DES1KO5R and DES1KO3f. After fusion with *HPH* cassette, a nested primer pair (DES1KO5F and DES1KO3R) was used for amplification of the final construct. Fungal protoplasts of the wild-type 70-15 were directly transformed with double-joint PCR product after purification. Protoplast generation and subsequent transformation were conducted by following the established procedures with slight modification. Initial identification of the gene disruption mutants was performed by PCR with the primer par of DES1KOSF and HPHF. Genomic DNA was isolated by the quick procedure described previously [70]. Candidates of gene disruption mutant were genetically purified by single conidia isolation. To confirm the disruption mutant, the genomic DNA of candidate strains was digested with *Bam*HI and Southern hybridization analysis was performed with 855 bp long 3′ flanking fragment (amplified with the primer pair of DES1KO3F and DES1KO3R) as a probe. For complementation of *Δdes1*, a 6 kb fragment carrying the *DES1* ORF and 1.4 kb of 5′ region (putative promoter and UTR) was amplified from wild-type genomic DNA using a primer pair of DES1_1400pF and DES1KO3R, and it cloned into pCR®-TOPO® 2.1 vector (Invitrogen, Carlsbad, CA, USA). The resulting plasmid was used for co-transforming *Δdes1* protoplasts with pII99 vector. The transformants were selected on TB3 agar medium amended with 800 ppm geneticin. After genetic purification by single conidium isolation, the presence of *DES1* ORF was checked by PCR amplification using a primer pair of DES1_QF and DES1_QR.

### Construction of DES1-eGFP vector

For fusion constructs, *Aspergillus nidulans TrpC* promoter (0.3 kb *Cla*I-*Hind*III fragment), eGFP coding sequence (0.7 kb *Hind*III-*Xba*I fragment) and DES1 ORF (4 kb *Spe*I or *Bam*HI fragment) were amplified by PCR from pSK1093, pSK2702 and genomic DNA from 70-15 using the primers shown in Table S2. Each primer contains a restriction enzyme site at its 5′ end to facilitate subsequent cloning. All three PCR products were isolated from gels using QIAquick spin columns and were cloned in pGEM-T Easy. All clones were verified by sequencing. Subsequently, the Pro*_TrpC_*-*DES1*-eGFP construct was inserted between the *Cla*I-*Xba*I sites of pGEM-3Zf (Promega, Madison, WI). The fusion constructs were co-transformed into wild-type 70-15 with pII99 that containing the geneticin resistant gene [83]. Three transformants per construct were selected and observed.

### Cell wall integrity test

For Nikkomycin Z sensitivity assays, conidia were incubated on slide glass with 100 µM of the drug, and germ tubes were observed after 2 hours. Protoplast production assay were performed as previously described [84] using 3-day-old mycelium (0.5 g) from CM liquid culture.

### Measurement of enzyme activity and detection of ferrous ion in extracellular culture filtrate

Enzyme activity was assayed using culture filtrate from 3-day-old CM liquid culture. Mycelia were completely removed by filtration and centrifugation (5,000 g at 4°C). For measurement of peroxidase and laccase activity, a reaction mixture (1 ml) containing 50 mM acetate buffer (pH 5.0) and 10 mM ABTS was mixed with the culture filtrate (200 µl) and incubated at 25°C for 5 minutes with or without 3mM of H_2_O_2_. Absorbance was evaluated at 420 nm.

Ferrous ion in the culture filtrate was measured with Bathophenanthroline disulfonate (BPS) color reaction. BPS was added to the culture filtrate (final concentration 1 mM), and the mixture was incubated for 3 hours at room temperature. Concentration of ferrous ion was monitored spectrophotometrically at 535 nm as BPS-Fe(II) complex formation. A standard curve was generated between color intensity and ferrous ion concentration by using standard solutions of varying concentrations (0–30 µM) of ferrous ion (OD_535_ = 0.213[Fe^2+^], R^2^ = 0.9995), and the absorbance at 535 nm was converted into ferrous ion concentration according to the standard curve.

### Bioinformatics

All sequence information used in this study was obtained from the online database CFGP ([38], http://cfgp.snu.ac.kr) which containing the latest annotated genome information of 59 fungi including *M. oryzae*. To identify *DES1* homologs, GeneBank (http://www.ncbi.nlm.nih.gov/BLAST) and CFGP database were searched using the BLAST algorithm [85]. Gene distribution analysis after *DES1* homolog search was performed automatically by the BLAST matrix program incorporated in CFGP. Sequence alignment using the ClustalW algorithm [86] and generation of bootstrapped phylogenetic trees were performed in CFGP. Results of InterPro Scan v12.0 [39], domain architecture visualization, SignalP v3.0 [40] and amino acid frequency analysis also were automatically provided from CFGP. Primers used in this study (Table S2) were designed using Primer Select™ program (DNASTAR Inc., Madison, USA) and commercially synthesized (BIONEER Corp., Daejeon, Korea).

## Supporting Information

Figure S1 Colony and conidia morphology of wild type, *Δdes1* and DES1^T-DNA^. (A) Morphology and color of 7-day old colonies of each strains on oatmeal agar media. (B) Morphology and color of conidia from the same cultures in panel A. Bars = 20 µm.
(1.86 MB PDF)

Figure S2 Amino acid sequence alignment of DES1p of *M. oryzae* with the homologs of other fungi. Amino acid sequences of DES1p of *M. oryzae* (MO), and the homologs of *C. globosum* (CG), *P. anserina* (PA), *N. crassa* (NC), *F. graminearum* (FG), *T. reesei* (TR), *B. cinerea* (BC), and *S. sclerotiorum* (SS) were aligned using ClustalW (Tompson et al., 1994). Identical amino acids are highlighted with a black background at 75% threshold.
(1.85 MB PDF)

Figure S3 Phylogenetic analysis of the *DES1* homologs. The *DES1* homologs comprised distinct phylogenetic clades according to the taxonomic distribution at the level of class. S, Sordariomycetes; D, Dothideomycetes; E, Eurotiomycetes; L, Leotiomycetes.
(0.03 MB PDF)

Figure S4 Identification of a *DES1* deletion mutant in *M. oryzae*. (A) The *DES1* deletion vector (4.5 kb) with the *HPH* cassette replaced the *DES1* ORF by double crossing over. Flanking genomic regions (white box) and the *Bgl*II restriction enzyme site are indicated. (B) Southern hybridization result. Total genomic DNA was digested with *Bam*HI, and the blot was probed with a DNA fragment of the 3′ flanking region indicated in panel A. Lane 1, 70-15 (wild type); Lane 2, *Δdes1*; Lane 3, E41 (an ectopic transformant). (C) Measured conidial size of the strains. Values are the mean±SD from >100 conidia of each strain that were measured using the Axiovision image analyzer. Columns with different letters are significantly different, as estimated using Tukey's HSD (Honestly Significant Differences) Test (*P* = 0.05).
(0.13 MB PDF)

Figure S5 Inhibition of ROS generation attenuates callose deposition and recovers IH development of *Δdes1* on onion epidermis. The onion epidermis was inoculated with conidial suspension (1×10^4^ conidia/ml) of the wild type, *Δdes1*, and DES1^T-DNA^ with or without diphenyleneiodonium (DPI) dissolved in DMSO. Samples were harvested and observed at 72 h after inoculation. Locations of appressoria are indicated with white arrowheads. TL, transmission light; RL, reflection light with a filter set with excitation at 470 nm and emission at 525 nm (UV excitation). Bar = 200 µm.
(0.68 MB PDF)

Figure S6 Re-introduction of wild type *DES1* allele to the *Δdes1* mutant complemented the IH development on rice and onion. The rice sheath and onion epidermis was inoculated with conidial suspension (1×10^4^ conidia/ml) of the wild type, *Δdes1*, and *Δdes1*::*DES1*. Samples were harvested and observed at 72 h after inoculation. Locations of appressoria (arrowheads) are indicated on rice sheath. Reflection light images of onion epidermis were observed with a filter set with excitation at 470 nm and emission at 525 nm (UV excitation). Bar = 200 µm.
(2.05 MB PDF)

Figure S7 Cell wall integrity tests using Nikkomycin Z and lysing enzyme. Sensitivity to chitin synthase inhibitor (Nikkomycin Z) and cell-wall-degrading enzyme (lysing enzyme) were tested for the wild type, *Δdes1*, and DES1^T-DNA^. (A) At 100 µM concentration, germination of all tested strains was not inhibited, and swellings at the basal appendix (arrowheads) are often observed. Bar = 20 µm. (B) Protoplast production by cell-wall-degrading enzyme. The released protoplast was quantified at regular time intervals.
(1.24 MB PDF)

Figure S8 Expression profiles of *M. oryzae* putative laccase-encoding genes in the *Δdes1* mutant. A combination of the phylogenetic tree, expression characteristics, and domain architecture of 16 putative laccases in the *M. oryzae* genome were displayed. The phylogenetic tree was generated by ClustalW sequence alignment with 1000 bootstrappings. The transcript levels of the putative laccase-encoding genes in the oxidative condition and/or in the *Δdes1* mutant are indicated. Relative abundance of transcript compared with standard condition (wild type, normal condition) is displayed as a white triangle (up-regulated) or an inverted black triangle (down-regulated). Triangles indicating more than 2.0 (fold change) are displayed as trapezoids by cutting the top of the triangle. Fold changes of the standard condition (1.0) are not shown. Up-regulated genes in the *Δdes1* mutant (more than 1.5 fold) were indicated in blue, and down-regulated genes in the *Δdes1* mutant (less than 0.6 fold) were indicated in red. The InterPro terms and signal peptides are indicated (see legend).
(0.07 MB PDF)

Figure S9 Comparison of ferrous ion concentrations between culture filtrates from the wild type, *Δdes1* and DES1^T-DNA^. Complex of BPS-Fe(II) was monitored by measurement of absorption at 535 nm using 3-day-old culture filtrates.
(0.06 MB PDF)

Figure S10 Expression Profiles of *DES1*. Expression of *DES1* during fungal developmental stages. Total RNA was isolated from conidia harvested from 3-day-old mycelia in liquid complete medium (vegetative growth), 10-day-old oatmeal agar medium (conidiation), 4 h-old germlings on hydrophobic surface of GelBond (germination), 24 h-old germlings on hydrophobic surface (appressorium formation), blast lesion enriched rice leaves (infectious growth), and 3-day-old liquid culture treated with 1 mM H_2_O_2_ for 30 minutes (oxidative stress). The transcriptional expression of *DES1* was analyzed by quantitative RT-PCR after synthesis of cDNA of each developmental RNA.
(0.04 MB PDF)

Table S1 *DES1* homologs are conserved strictly in Subphylum Pezizomycotina.
(0.01 MB PDF)

Table S2 Primers used in this study.
(0.01 MB PDF)
