# Supplementary material for: A Novel Pathogenicity Gene Is Required in the Rice Blast Fungus to Suppress the Basal Defenses of the Host
Source: PLoS Pathog. 2009 Apr 24;5(4):e1000401. doi: 10.1371/journal.ppat.1000401 (PMC2668191; doi:10.1371/journal.ppat.1000401)
Supplement: Figure S1 — Colony and conidia morphology of wild type, Δdes1 and DES1T-DNA. (A) Morphology and color of 7-day old colonies of each strains on oatmeal agar media. (B) Morphology and color of conidia from the same cultures in panel A. Bars = 20 µm. (1.86 MB PDF) [file ppat.1000401.s001.pdf]

Figure S1

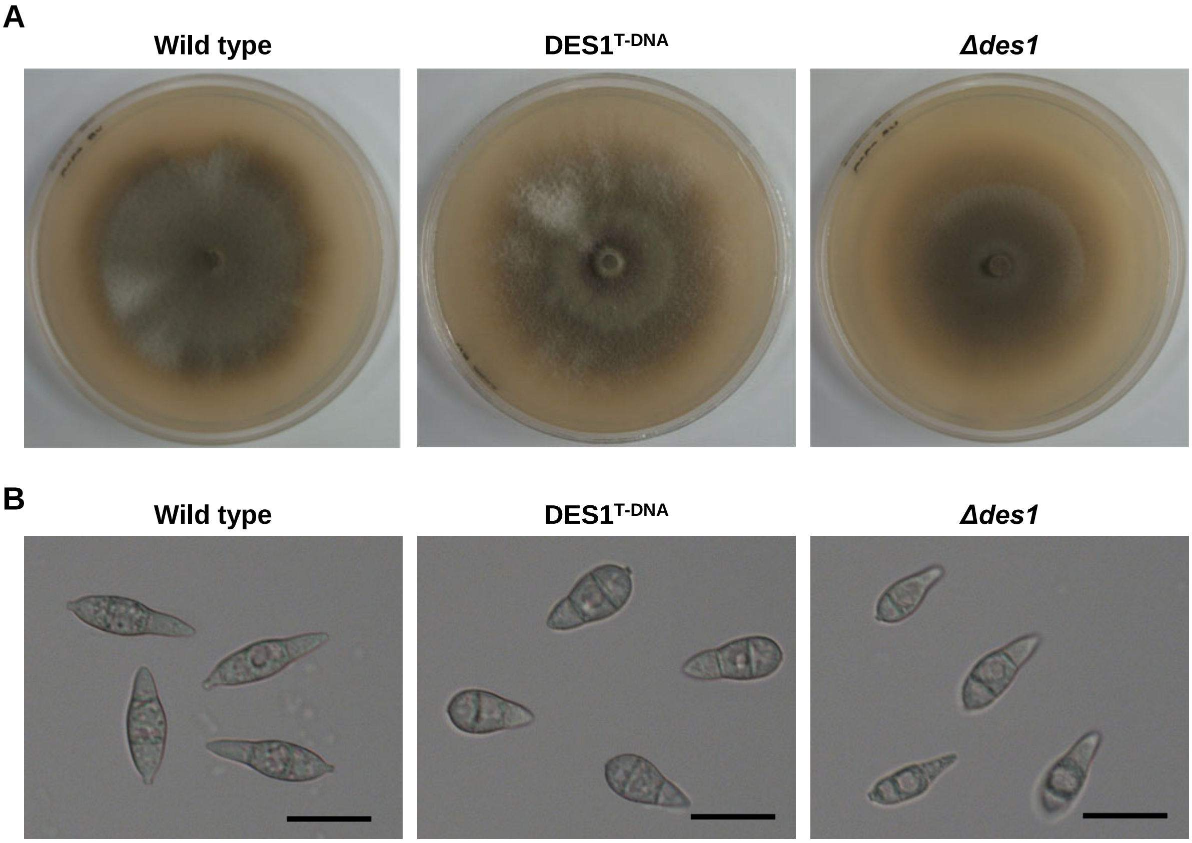

Figure S1. Colony and conidia morphology of wild type, *Δdes1*and DES1$^{\text{T-DNA}}$.

(A) Morphology and color of 7-day old colonies of each strains on oatmeal agar media.

(B) Morphology and color of conidia from the same cultures in panel A. Bars = 20 μm.
