# Supplementary figures and images for: A Novel Pathogenicity Gene Is Required in the Rice Blast Fungus to Suppress the Basal Defenses of the Host
Source: PLoS Pathog. 2009 Apr 24;5(4):e1000401. doi: 10.1371/journal.ppat.1000401 (PMC2668191; doi:10.1371/journal.ppat.1000401)

Figure S2

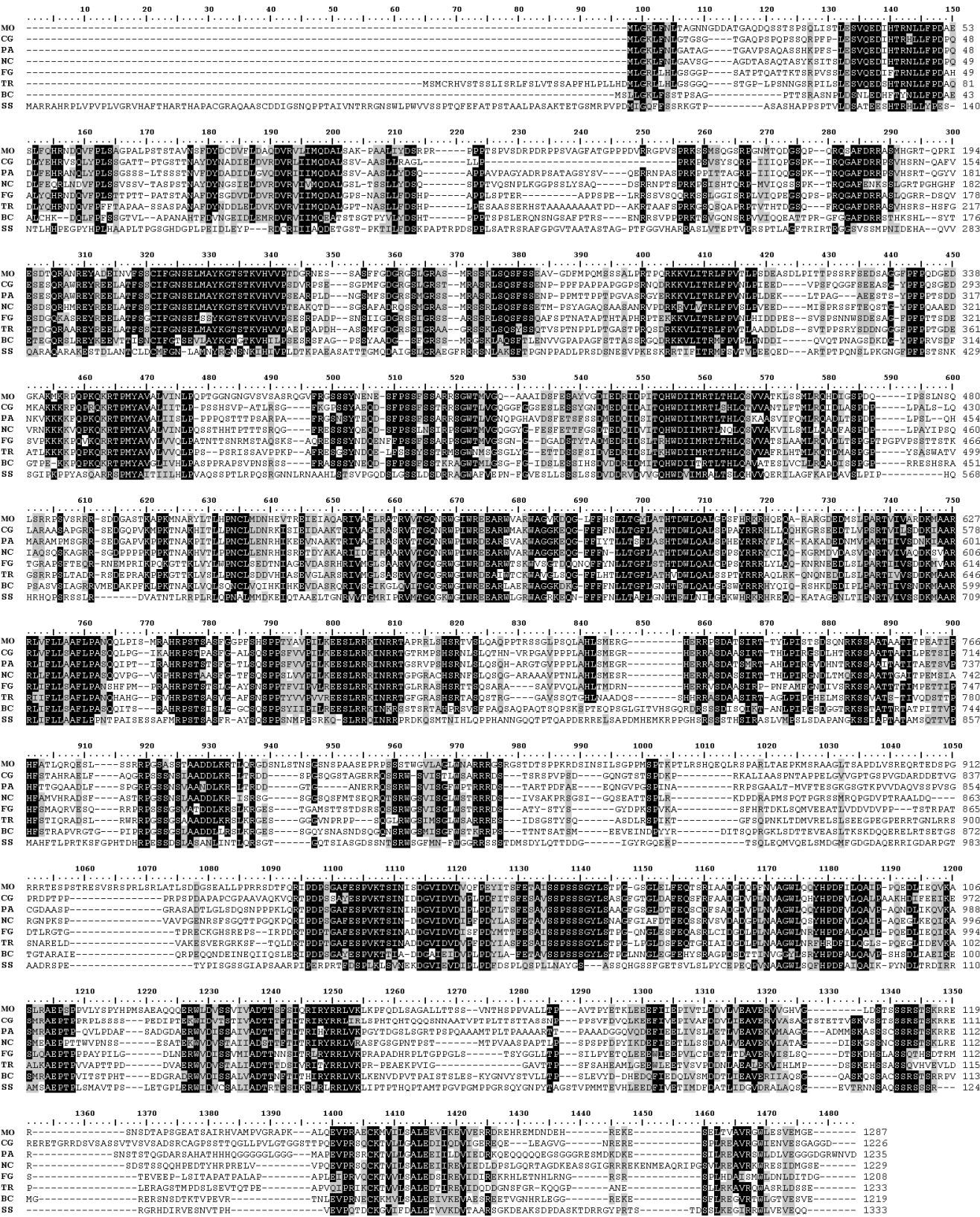

Supplement: Figure S2 — Amino acid sequence alignment of DES1p of M. oryzae with the homologs of other fungi. Amino acid sequences of DES1p of M. oryzae (MO), and the homologs of C. globosum (CG), P. anserina (PA), N. crassa (NC), F. graminearum (FG), T. reesei (TR), B. cinerea (BC), and S. sclerotiorum (SS) were aligned using ClustalW (Tompson et al., 1994). Identical amino acids are highlighted with a black background at 75% threshold. (1.85 MB PDF) [file ppat.1000401.s002.pdf]
