# Supplementary material for: A Novel Pathogenicity Gene Is Required in the Rice Blast Fungus to Suppress the Basal Defenses of the Host
Source: PLoS Pathog. 2009 Apr 24;5(4):e1000401. doi: 10.1371/journal.ppat.1000401 (PMC2668191; doi:10.1371/journal.ppat.1000401)
Supplement: Figure S3 — Phylogenetic analysis of the DES1 homologs. The DES1 homologs comprised distinct phylogenetic clades according to the taxonomic distribution at the level of class. S, Sordariomycetes; D, Dothideomycetes; E, Eurotiomycetes; L, Leotiomycetes. (0.03 MB PDF) [file ppat.1000401.s003.pdf]

Figure S3

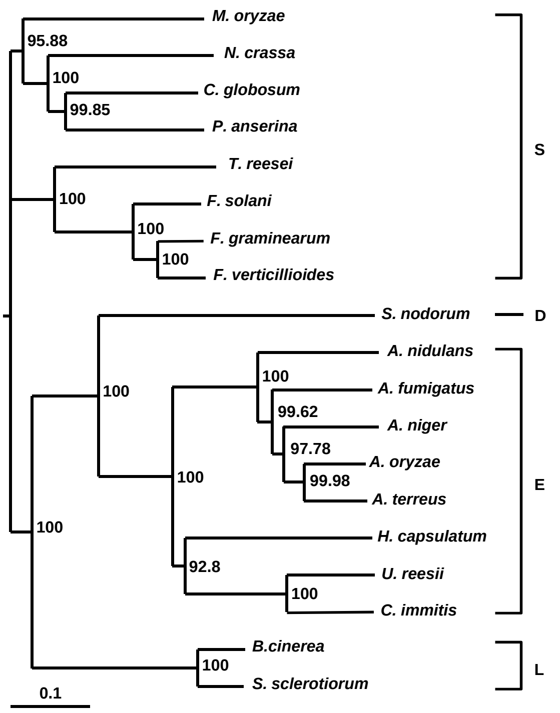

Figure S3. Phylogenetic analysis of the *DES1* homologs.

The *DES1* homologs comprised distinct phylogenetic clades according to the taxonomic distribution at the level of class. S, Sordariomycetes; D, Dothideomycetes; E, Eurotiomycetes; L, Leotiomycetes.
