# Supplementary material for: A Novel Pathogenicity Gene Is Required in the Rice Blast Fungus to Suppress the Basal Defenses of the Host
Source: PLoS Pathog. 2009 Apr 24;5(4):e1000401. doi: 10.1371/journal.ppat.1000401 (PMC2668191; doi:10.1371/journal.ppat.1000401)
Supplement: Figure S4 — Identification of a DES1 deletion mutant in M. oryzae. (A) The DES1 deletion vector (4.5 kb) with the HPH cassette replaced the DES1 ORF by double crossing over. Flanking genomic regions (white box) and the BglII restriction enzyme site are indicated. (B) Southern hybridization result. Total genomic DNA was digested with BamHI, and the blot was probed with a DNA fragment of the 3′ flanking region indicated in panel A. Lane 1, 70-15 (wild type); Lane 2, Δdes1; Lane 3, E41 (an ectopic transformant). (C) Measured conidial size of the strains. Values are the mean±SD from >100 conidia of each strain that were measured using the Axiovision image analyzer. Columns with different letters are significantly different, as estimated using Tukey's HSD (Honestly Significant Differences) Test (P = 0.05). (0.13 MB PDF) [file ppat.1000401.s004.pdf]

Figure S4

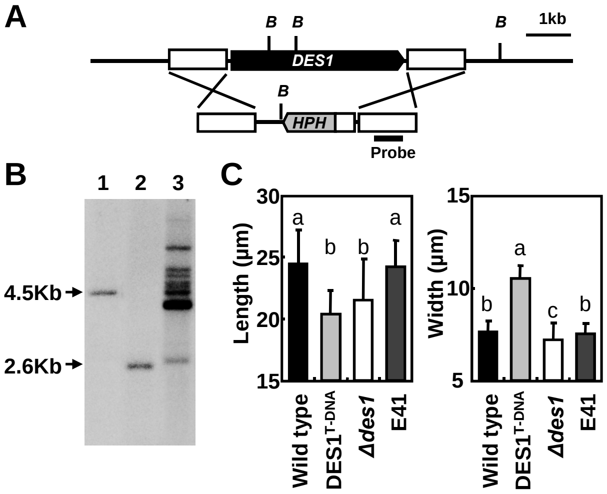

Figure S4. Identification of a *DES1* deletion mutant in *M. oryzae.*

(A) The *DES1* deletion vector (4.5 kb) with the *HPH* cassette replaced the *DES1* ORF by double crossing over. Flanking genomic regions (white box) and the *Bgl*II restriction enzyme site are indicated. **(B)** Southern hybridization result. Total genomic DNA was digested with *Bam*HI, and the blot was probed with a DNA fragment of the 3’ flanking region indicated in panel A. Lane 1, 70-15 (wild type); Lane 2, *Δdes1*; Lane 3, E41 (an ectopic transformant).
(C) Measured conidial size of the strains. Values are the mean ± SD from > 100 conidia of each strain that were measured using the Axiovision image analyzer. Columns with different letters are significantly different, as estimated using Tukey's HSD (Honestly Significant Differences) Test ($P = 0.05$).
