# Supplementary material for: A Novel Pathogenicity Gene Is Required in the Rice Blast Fungus to Suppress the Basal Defenses of the Host
Source: PLoS Pathog. 2009 Apr 24;5(4):e1000401. doi: 10.1371/journal.ppat.1000401 (PMC2668191; doi:10.1371/journal.ppat.1000401)
Supplement: Figure S5 — Inhibition of ROS generation attenuates callose deposition and recovers IH development of Δdes1 on onion epidermis. The onion epidermis was inoculated with conidial suspension (1×10^4^ conidia/ml) of the wild type, Δdes1, and DES1T-DNA with or without diphenyleneiodonium (DPI) dissolved in DMSO. Samples were harvested and observed at 72 h after inoculation. Locations of appressoria are indicated with white arrowheads. TL, transmission light; RL, reflection light with a filter set with excitation at 470 nm and emission at 525 nm (UV excitation). Bar = 200 µm. (0.68 MB PDF) [file ppat.1000401.s005.pdf]

Figure S5

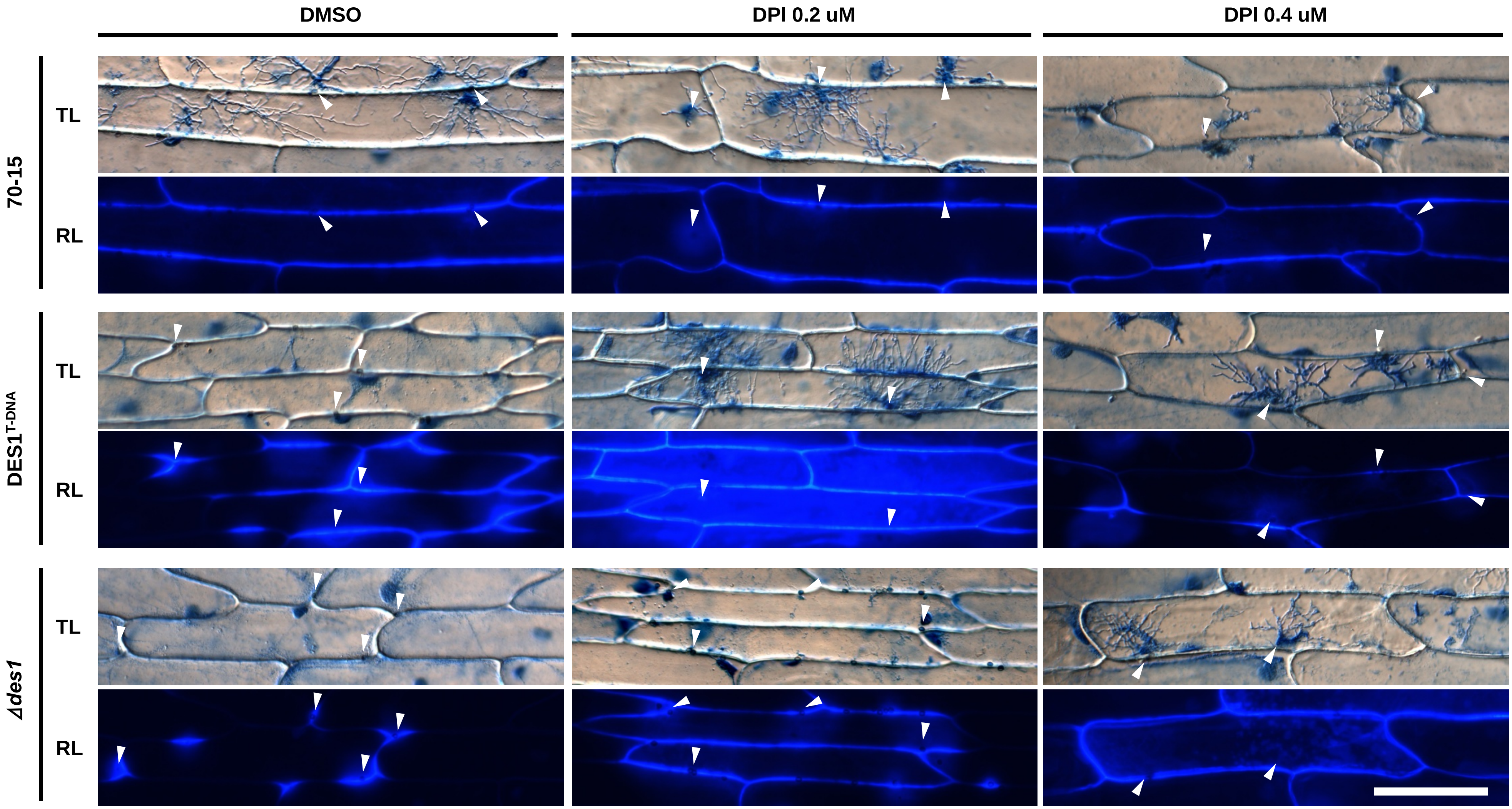

Figure S5. Inhibition of ROS generation attenuates callose deposition and recovers IH development of *Δdes1* on onion epidermis.

The onion epidermis was inoculated with conidial suspension ($1x10^4$ conidia/ml) of the wild type, *Δdes1*, and $DES1^{T\text{-}DNA}$ with or without diphenyleneiodonium (DPI) dissolved in DMSO. Samples were harvested and observed at 72 h after inoculation. Locations of appressoria are indicated with white arrowheads. TL, transmission light; RL, reflection light with a filter set with excitation at 470 nm and emission at 525 nm (UV excitation). Bar = 200 µm.
