# Supplementary material for: A Novel Pathogenicity Gene Is Required in the Rice Blast Fungus to Suppress the Basal Defenses of the Host
Source: PLoS Pathog. 2009 Apr 24;5(4):e1000401. doi: 10.1371/journal.ppat.1000401 (PMC2668191; doi:10.1371/journal.ppat.1000401)
Supplement: Figure S6 — Re-introduction of wild type DES1 allele to the Δdes1 mutant complemented the IH development on rice and onion. The rice sheath and onion epidermis was inoculated with conidial suspension (1×10^4^ conidia/ml) of the wild type, Δdes1, and Δdes1::DES1. Samples were harvested and observed at 72 h after inoculation. Locations of appressoria (arrowheads) are indicated on rice sheath. Reflection light images of onion epidermis were observed with a filter set with excitation at 470 nm and emission at 525 nm (UV excitation). Bar = 200 µm. (2.05 MB PDF) [file ppat.1000401.s006.pdf]

Figure S6

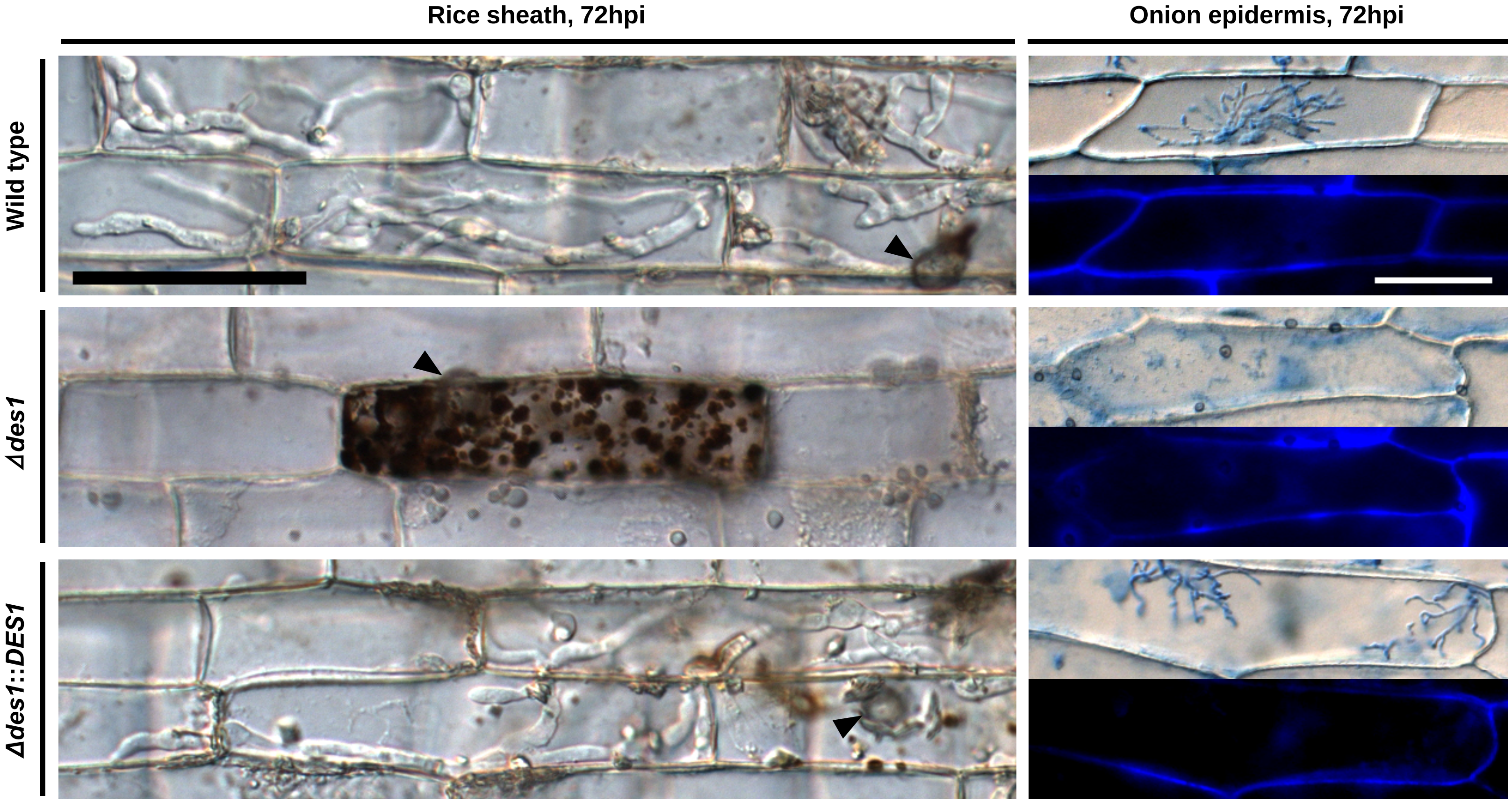

Figure S6. Re-introduction of wild type DES1 allele to the *Δ*des1 mutant complemented the IH development on rice and onion.
The rice sheath and onion epidermis was inoculated with conidial suspension (1x10^4 conidia/ml) of the wild type, *Δdes1*, and *Δdes1*:DES1. Samples were harvested and observed at 72 h after inoculation. Locations of appressoria (arrowheads) are indicated on rice sheath. Reflection light images of onion epidermis were observed with a filter set with excitation at 470 nm and emission at 525 nm (UV excitation). Bar = 200 µm.
