# Supplementary material for: A Novel Pathogenicity Gene Is Required in the Rice Blast Fungus to Suppress the Basal Defenses of the Host
Source: PLoS Pathog. 2009 Apr 24;5(4):e1000401. doi: 10.1371/journal.ppat.1000401 (PMC2668191; doi:10.1371/journal.ppat.1000401)
Supplement: Figure S7 — Cell wall integrity tests using Nikkomycin Z and lysing enzyme. Sensitivity to chitin synthase inhibitor (Nikkomycin Z) and cell-wall-degrading enzyme (lysing enzyme) were tested for the wild type, Δdes1, and DES1T-DNA. (A) At 100 µM concentration, germination of all tested strains was not inhibited, and swellings at the basal appendix (arrowheads) are often observed. Bar = 20 µm. (B) Protoplast production by cell-wall-degrading enzyme. The released protoplast was quantified at regular time intervals. (1.24 MB PDF) [file ppat.1000401.s007.pdf]

Figure S7

A

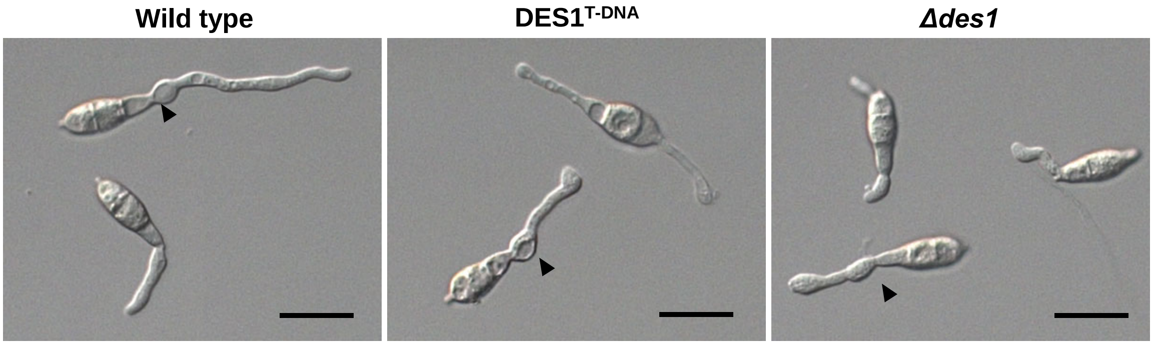

B

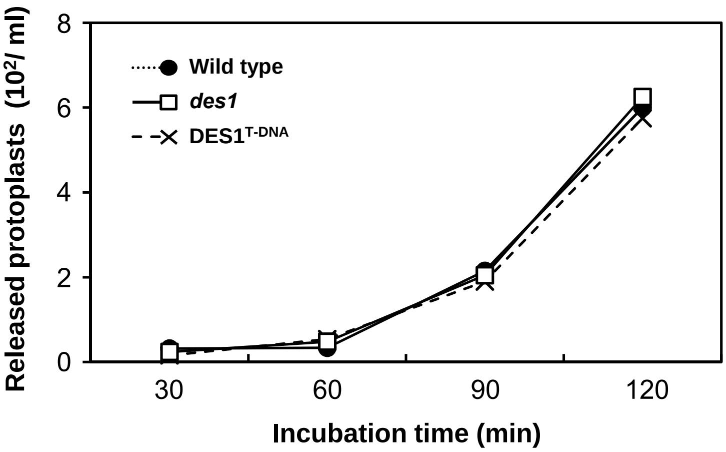

Figure S7. Cell wall integrity tests using Nikkomycin Z and lysing enzyme.

Sensitivity to chitin synthase inhibitor (Nikkomycin Z) and cell-wall-degrading enzyme (lysing enzyme) were tested for the wild type, *Δdes1*, and DES1$^{T\text{-}DNA}$.

(A) At 100 μM concentration, germination of all tested strains was not inhibited, and swellings at the basal appendix (arrowheads) are often observed. Bar = 20 μm.

(B) Protoplast production by cell-wall-degrading enzyme. The released protoplast was quantified at regular time intervals.
