# Supplementary material for: A Novel Pathogenicity Gene Is Required in the Rice Blast Fungus to Suppress the Basal Defenses of the Host
Source: PLoS Pathog. 2009 Apr 24;5(4):e1000401. doi: 10.1371/journal.ppat.1000401 (PMC2668191; doi:10.1371/journal.ppat.1000401)
Supplement: Figure S8 — Expression profiles of M. oryzae putative laccase-encoding genes in the Δdes1 mutant. A combination of the phylogenetic tree, expression characteristics, and domain architecture of 16 putative laccases in the M. oryzae genome were displayed. The phylogenetic tree was generated by ClustalW sequence alignment with 1000 bootstrappings. The transcript levels of the putative laccase-encoding genes in the oxidative condition and/or in the Δdes1 mutant are indicated. Relative abundance of transcript compared with standard condition (wild type, normal condition) is displayed as a white triangle (up-regulated) or an inverted black triangle (down-regulated). Triangles indicating more than 2.0 (fold change) are displayed as trapezoids by cutting the top of the triangle. Fold changes of the standard condition (1.0) are not shown. Up-regulated genes in the Δdes1 mutant (more than 1.5 fold) were indicated in blue, and down-regulated genes in the Δdes1 mutant (less than 0.6 fold) were indicated in red. The InterPro terms and signal peptides are indicated (see legend). (0.07 MB PDF) [file ppat.1000401.s008.pdf]

Figure S8

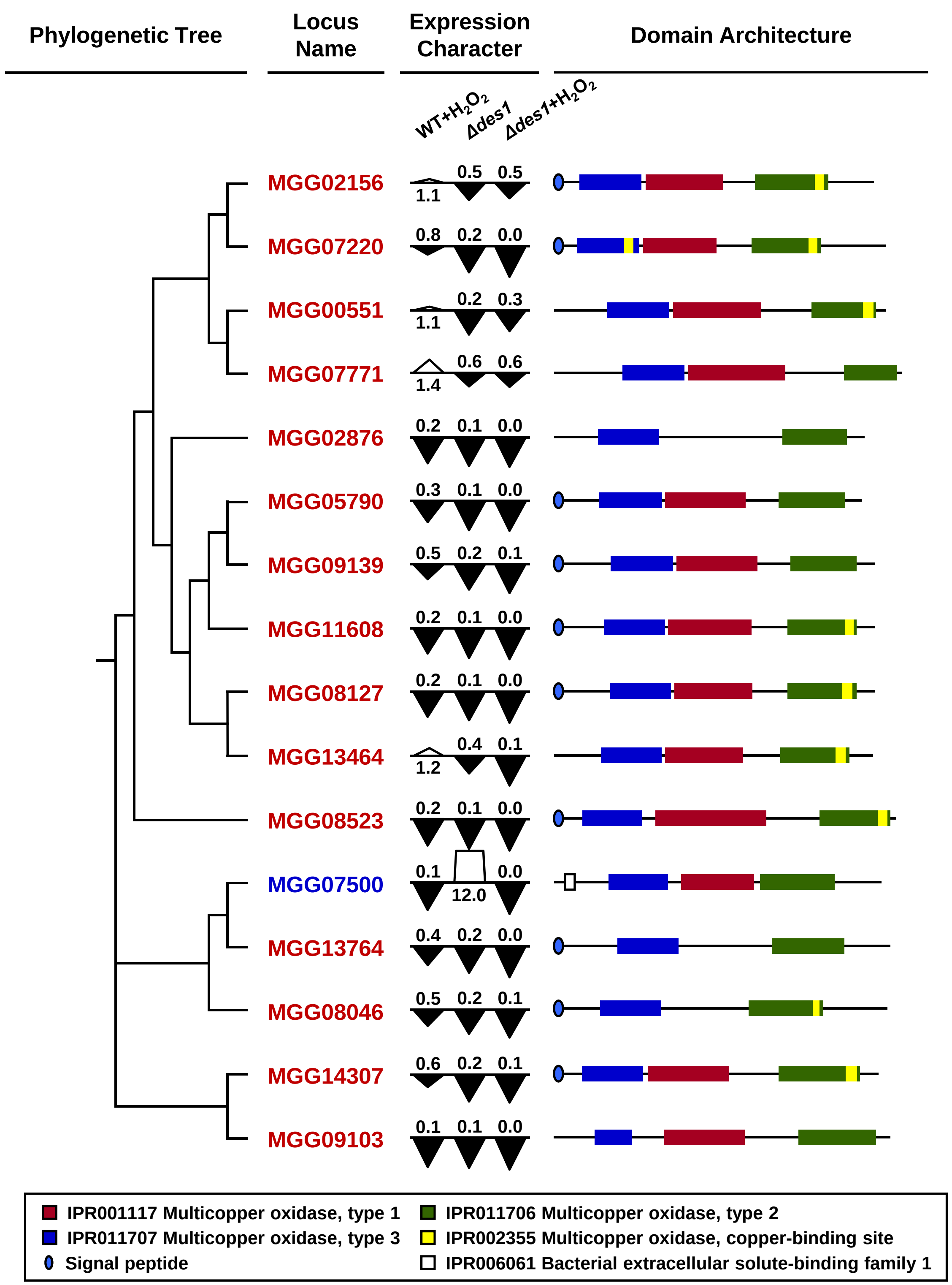

Figure S8. Expression profiles of *M. oryzae* putative laccase-encoding genes in the *Δdes1* mutant.

A combination of the phylogenetic tree, expression characteristics, and domain architecture of 16 putative laccases in the *M. oryzae* genome were displayed. The phylogenetic tree was generated by ClustalW sequence alignment with 1000 bootstrappings. The transcript levels of the putative laccase-encoding genes in the oxidative condition and/or in the *Δdes1* mutant are indicated. Relative abundance of transcript compared with standard condition (wild type, normal condition) is displayed as a white triangle (up-regulated) or an inverted black triangle (down-regulated). Triangles indicating more than 2.0 (fold change) are displayed as trapezoids by cutting the top of the triangle. Fold changes of the standard condition (1.0) are not shown. Up-regulated genes in the *Δdes1* mutant (more than 1.5 fold) were indicated in blue, and down-regulated genes in the *Δdes1* mutant (less than 0.6 fold) were indicated in red. The InterPro terms and signal peptides are indicated (see legend).
