# Supplementary material for: A Novel Pathogenicity Gene Is Required in the Rice Blast Fungus to Suppress the Basal Defenses of the Host
Source: PLoS Pathog. 2009 Apr 24;5(4):e1000401. doi: 10.1371/journal.ppat.1000401 (PMC2668191; doi:10.1371/journal.ppat.1000401)
Supplement: Figure S9 — Comparison of ferrous ion concentrations between culture filtrates from the wild type, Δdes1 and DES1T-DNA. Complex of BPS-Fe(II) was monitored by measurement of absorption at 535 nm using 3-day-old culture filtrates. (0.06 MB PDF) [file ppat.1000401.s009.pdf]

Figure S9

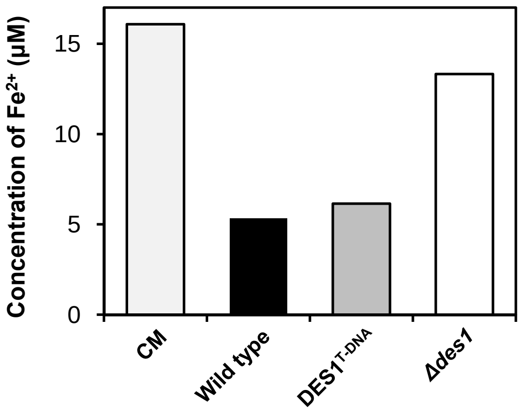

Figure S9. **Comparison of ferrous ion concentrations between culture filtrates from the wild type, *Δdes1* and DES1$^{T\text{-}DNA}$.**

Complex of BPS-Fe(II) was monitored by measurement of absorption at 535nm using 3-day-old culture filtrates.
