# Supplementary material for: A Novel Pathogenicity Gene Is Required in the Rice Blast Fungus to Suppress the Basal Defenses of the Host
Source: PLoS Pathog. 2009 Apr 24;5(4):e1000401. doi: 10.1371/journal.ppat.1000401 (PMC2668191; doi:10.1371/journal.ppat.1000401)
Supplement: Figure S10 — Expression Profiles of DES1. Expression of DES1 during fungal developmental stages. Total RNA was isolated from conidia harvested from 3-day-old mycelia in liquid complete medium (vegetative growth), 10-day-old oatmeal agar medium (conidiation), 4 h-old germlings on hydrophobic surface of GelBond (germination), 24 h-old germlings on hydrophobic surface (appressorium formation), blast lesion enriched rice leaves (infectious growth), and 3-day-old liquid culture treated with 1 mM H2O2 for 30 minutes (oxidative stress). The transcriptional expression of DES1 was analyzed by quantitative RT-PCR after synthesis of cDNA of each developmental RNA. (0.04 MB PDF) [file ppat.1000401.s010.pdf]

Figure S10

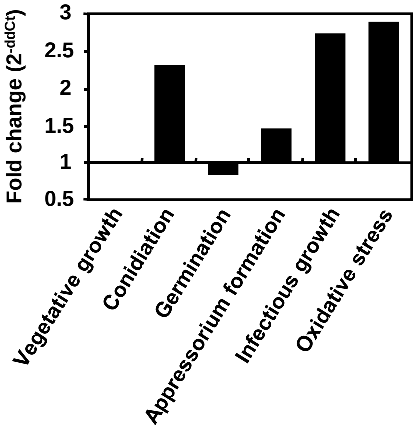

Figure S10. Expression Profiles of *DES1*.

Expression of *DES1* during fungal developmental stages. Total RNA was isolated from conidia harvested from 3-day-old mycelia in liquid complete medium (vegetative growth), 10-day-old oatmeal agar medium (conidiation), 4 h-old germlings on hydrophobic surface of GelBond (germination), 24 h-old germlings on hydrophobic surface (appressorium formation), blast lesion enriched rice leaves (infectious growth), and 3-day-old liquid culture treated with 1 mM H_2O_2 for 30 minutes (oxidative stress). The transcriptional expression of *DES1* was analyzed by quantitative RT-PCR after synthesis of cDNA of each developmental RNA.
