# Supplementary material for: A Novel Pathogenicity Gene Is Required in the Rice Blast Fungus to Suppress the Basal Defenses of the Host
Source: PLoS Pathog. 2009 Apr 24;5(4):e1000401. doi: 10.1371/journal.ppat.1000401 (PMC2668191; doi:10.1371/journal.ppat.1000401)
Supplement: Table S1 — DES1 homologs are conserved strictly in Subphylum Pezizomycotina. (0.01 MB PDF) [file ppat.1000401.s011.pdf]

Table S1. *DES1* homologs are conserved strictly in Subphylum Pezizomycotina.

Phylum	Subphylum	Species	Locus Name	Protein Length	E-value	Count	Sequence Identity
**Oomycota**		*Phytophthora infestans*	None				
		Phytophthora ramorum	None				
		Phytophthora sojae	None				
**Ascomycota**	**Pezizomycotina**	*Magnaporthe oryzae*	*DES1* (MGG_04163.6)	1287	0	1	100%
		Chaetomium globosum	CHG04628.1	1226	0	1	52.29%
		Podospora anserina	Pa_6_2820	1236	0	1	51.59%
		Neurospora crassa	NCU07707.2	1229	0	1	50.18%
		Fusarium oxysporum	FOXG_08291	1210	0	1	50.07%
		Fusarium verticillioides	fver-2.8-g98.1	1210	0	1	50.03%
		Fusarium graminearum	FGSG_09765.3	1209	0	1	49.80%
		Fusarium solani	fgenesh1_pm.sca_18_chr2_1_000045[illegible]	1202	0	1	49.80%
		Trichoderma reesei	33195	1233	0	1	48.30%
		Botrytis cinerea	BC1G_03084.1	1219	0	1	44.74%
		Sclerotinia sclerotiorum	SS1G_08359.1	1221	0	1	44.32%
		Stagonospora nodorum	SNU08688.1	1333	1.0E-159	1	33.01%
		Aspergillus terreus	ATEG_06731.1	1206	1.0E-137	1	31.42%
		Aspergillus oryzae	AO090001000651	1199	1.0E-132	1	31.39%
		Histoplasma capsulatum	HCAG_00936.1	1196	1.0E-129	1	30.61%
		Coccidioides immitis	CIMG_00711.2	1215	1.0E-129	1	30.46%
		Aspergillus fumigatus	Afu2g05410	1208	1.0E-126	1	30.23%
		Uncinocarpus reesii	UREG_00701.1	1216	1.0E-130	1	29.91%
		Aspergillus nidulans	AN7504.3	1168	1.0E-124	1	29.51%
		Aspergillus niger	estExt_fgenesh1_pg.C_40696	1211	1.0E-126	1	29.02%
		Mycosphaerella graminicola	None				
	**Saccharomycotina**	*Candida albicans*	None				
		Candida glabrata	None				
		Candida guilliermondii	None				
		Candida lusitaniae	None				
		Candida tropicalis	None				
		Eremothecium gossypii	None				
		Kluyveromyces lactis	None				
		Kluyveromyces waltii	None				
		Lodderomyces elongisporus	None				
		Pichia stipitis	None				
		Saccharomyces bayanus	None				
		Saccharomyces castellii	None				
		Saccharomyces cerevisiae 288C 2007	None				
		Saccharomyces cerevisiae RM11	None				
		Saccharomyces cerevisiae YJM789	None				
		Saccharomyces kluyveri	None				
		Saccharomyces kudriavzevii	None				
		Saccharomyces mikatae	None				
		Saccharomyces paradoxus	None				
		Yarrowia lipolytica	None				
	**Taphrinomycotina**	*Pneumocystis carinii*	None				
		Schizosaccharomyces japonicus	None				
		Schizosaccharomyces pombe	None				
**Basidiomycota**		*Coprinus cinereus 1*	None				
		Cryptococcus neoformans serotype B	None				
		Cryptococcus neoformans serotype D	None				
		Laccaria bicolor	None				
		Phanerochaete chrysosporium	None				
		Ustilago maydis	None				
**Chitridiomycota**		*Batrachochytrium dendrobatidis*	None				
**Microsporidia**		*Antonospora locustae*	None				
		Encephalitozoon cuniculi	None				
**Pucciniomycotina**		*Sporobolomyces roseus*	None				
**Zygomycota**		*Phycomyces blakesleeanus*	None				
		Rhizopus oryzae	None				
