# Supplementary material for: A Novel Pathogenicity Gene Is Required in the Rice Blast Fungus to Suppress the Basal Defenses of the Host
Source: PLoS Pathog. 2009 Apr 24;5(4):e1000401. doi: 10.1371/journal.ppat.1000401 (PMC2668191; doi:10.1371/journal.ppat.1000401)
Supplement: Table S2 — Primers used in this study. (0.01 MB PDF) [file ppat.1000401.s012.pdf]

Table S2. Primers used in this study.

Name	Sequences (5’-3’)	References
4163TF	GAGCGAGCGAGTATGATGTAG	
4163TR	CAAGTTGCGGGTATGTATGTC	
CATA_QF *	GGTGGACGCCGTGTACGT	(Skamnioti et al., 2007)
CATA_QR *	CCGTTCTTGGACAATGTCTTGA	(Skamnioti et al., 2007)
CATB_QF *	GTAAGCGCCCAGACTACAAG	
CATB_QR *	GACATGGCCCAGAAAAGAG	
CCPB_QF *	GGGATGGTCCTAAGCAGTACGTC	(Skamnioti et al., 2007)
CCPB_QR *	TGTCCTCGATGAGGCACATG	(Skamnioti et al., 2007)
CPXA_QF *	GTGGCCTATCAAGCAGAAGTAT	
CPXA_QR *	TCGTTGCCCGAAGAGTATC	
CPXB_QF *	CGTGGCGGTGGTGGAAT	(Skamnioti et al., 2007)
CPXB_QR *	TGATGGGCCAAATCAGCC	(Skamnioti et al., 2007)
CYPF_qRT *	GCCTAACGTTTTCTTCGACATTTC	
CYPR_qRT *	GTTCTCGTCGGCAAACTTCTC	
DES1_0F	ATGCTCGGGAAGCTTTTCAA	
DES1_1F	GCGAGTATGCCGATGAGAT	
DES1_2F	CCTTGCTGCGTTTCTACCT	
DES1_3R	GGTGGATGTGCCTTTGTAG	
DES1_4R	GTGGTGGTATGGCCTGTAGTA	
DES1_6R	AGATGGGCGAGTTGAGAAG	
DES1_QF	AGGCGAAAATGAAGAGACC	
DES1_QR	CGCAGAGGAGAAAGAAGATG	
DES1KO3F	CCTTCAATATCATCTTCTGTCGAAATGGGGACGAGCAGTT	
DES1KO3R	TCCCAGCGAGTAGTCAAGTG	
DES1KO5F	GAGTGCAGGCTTGATTTCTT	
DES1KO5R	GTTTTGGTGGCTGGCTTACGCACAGGTACACTTGTTTAGA	
DES1KOSF	TCCTGTTCGTCTTTACCTGAG	
DES1KOSR	ACCTAGCTGCTGACAAATCC	
HPHF	TTCCGGAAGTGCTTGACATT	
HPHR	TTCTACACAGCCATCGGTCC	
HygB-f	CTCTAAACAAGTGTACCTGTGC	(Kim et al., 2005a)
HygB-r	CGACAGAAGATGATATTGAAGG	(Kim et al., 2005a)
RB3	CCCTTCCCAACAGTTGCGCA	(Mullins et al., 2001)
LB3	GAATTAATTCGGCGTTAATTCAGT	(Mullins et al., 2001)
MGG00461_QF	GGGAGACTTTTGCTTGGTA	
MGG00461_QR	CTTTTCTTTGCGGACTGCT	
MGG07554_QF	ACTTCGCTTGTGTTCTTCTC	
MGG07554_QR	AGGCGTTCCGTGTAGTGAC	
MGG07574_QF	GGAGCAGATGTTCACCTTTAT	
MGG07574_QR	GGGCAGCGATCTTTGTAGT	
MGG07790_QF	CAGAGCAGAACAAGCAGGTC	
MGG07790_QR	GAGTCGAGGTGGTGGTTCT	
MGG07871_QF	CAAGTTCGAAAAGTCCATCTG	
MGG07871_QR	AAAAATAACCTTCAACCACTCC	
MGG08200_QF	GTTTGCATACTTTGGGACAGA	
MGG08200_QR	GTGGGGATTGGAGGTAGACT	
MGG09398_QF	CGTTTTCATGGTGGGAGTTT	
MGG09398_QR	GTCACGGGGAAGTTGTTGTT	
MGG10368_QF	AGGTGGGTCAAGGTGTATCG	
MGG10368_QR	AATCTTGCCTTGCTTTTTAG	
MGG10859_QF	AACCATTGGGCAGTCTACC	
MGG10859_QR	CTCCCCGATACCAGTCAAT	
MGG10877_QF	AGTGCCCGTCAATGGTAAC	
MGG10877_QR	TCACAAATAGGCACAAGAGGT	
MGG11608_QF	TACAAGCGCTACTGCCAGAC	

(continue)

Name	Sequences (5’ - 3’)	References
MGG11608_QR	CACCAGGAACACCATCATACTC	
MGG11849_QF	CAACAGTCTCGCCAACCAC	
MGG11849_QR	CTGCAGTCGGAAGAAGAGC	
MGG13239_QF	GATCTCCAGCGTTCTCTTTTAC	
MGG13239_QR	CTTTAGCAGTCCGTCCTTCA	
MGG02156_QF	ACCTGATAGCGGGATTTTG	
MGG02156_QR	GGCCGACGTAAGTGATGTT	
MGG00551_QF	TCGAGGCTGATGGGATTCT	
MGG00551_QR	ATGCTGCCGTAGGGATTATG	
MGG02876_QF	TGGCCCAATGGTCTTCTAT	
MGG02876_QR	TGCCGTTGATCAGGTTGTT	
MGG05790_QF	CTCGCTTGACAACCACACC	
MGG05790_QR	CCGAGGCTTGACCGTAGTAG	
MGG07220_QF	CGCTACGACGTGCTCATCT	
MGG07220_QR	GTCGCCAACAACATCGTAAG	
MGG07500_QF	GTTGGGCAGAGGATTTGAC	
MGG07500_QR	AGAGTGCCGTTGCTGTTGT	
MGG07771_QF	CCTACCCCGACAACATCAT	
MGG07771_QR	GCCGTCCATCTCCACTATC	
MGG08046_QF	TTACAACGACCCCAACTTCAT	
MGG08046_QR	CCTTTCCCGCTGTCATTCT	
MGG08127_QF	CAGTACATCGCGGAGAAAAA	
MGG08127_QR	GACGGGGTTATTGAAGTTGAG	
MGG08523_QF	AAGTGGAAGGCGGAACAGTA	
MGG08523_QR	GCCAGTCGGAGAGGATGAT	
MGG09102_QF	AGCCCAGACGAGAAGATACAA	
MGG09102_QR	ATAAATGCCATGCCAGTGC	
MGG09103_QF	CAGTCACCATGCCCAACAC	
MGG09103_QR	TAGAGCCCCTGCGAGATTC	
MGG09139_QF	TGATCAACGGTACCAATGTCTA	
MGG09139_QR	CCTATGCCGATGCTTACCA	
MGG13464_QF	AACCTTGAGATTAACGGAACAT	
MGG13464_QR	TGAGAGTGGTACCAAGAAGTGC	
MGG13764_QF	GTCAACAACGCTGCTCTCC	
MGG13764_QR	GGGGTCCGTGATGATGTAG	
MGG14307_QF	GGCCAGTACCAACAAGACAA	
MGG14307_QR	AAGGGCTGCAGGGTAAACT	
MGG04162_QF	TCGAAACCAGAAGACCAGA	
MGG04162_QR	AATGCCATCGCTAAACTCA	
Rice_actin_QF	CTTCAACACCCCTGCTATG	
Rice_actin_QR	CCGTTGTGGTGAATGAGTAA	
Rice_PBZ1_QF	CTACTATGGCATGCTCAAGAT	
Rice_PBZ1_QR	ATAGAAAGGCACATAAACACAA	
Rice_PR1a_QF	TCTTCATCACCTGCAACTACTC	
Rice_PR1a_QR	ATTCATCGGATTTATTCTCACC	
TV1	ACTAGAACCGGAGACATTACG	
DES1_3864F	ACACTAGTATGCTCGGGAAGCTTTTCAACCTG	
DES1_3864R	ACTCTAGATTCTCCCATCTCGACACTCTCCAAC	
DES1_1400pF	CTCGTGGTAATGCGATTTGATTCCC	
GFP 720F	ACTCTAGAATGGTGAGCAAGGGCGAGG	
GFP 720R	ACGCGGCCGCTTACTTGTACAGCTCGTCCATGCCG	
TrpC 360F	ACATCGATAGAAGATGACATTGAAGGAGCACTTTTTGGG	
TrpC 360R	ACACTAGTATGCTTGGGTAGAATAGGTAAGTCAGATTGA	

* CATA (MGG100061), CATB (MGG06442), CPXA (MGG04337), CPXB (MGG09834), CCPB (MGG04545), CYP (MGG10447)
